# Burden of Depressive Disorders by Country, Sex, Age, and Year: Findings from the Global Burden of Disease Study 2010

**DOI:** 10.1371/journal.pmed.1001547

**Published:** 2013-11-05

**Authors:** Alize J. Ferrari, Fiona J. Charlson, Rosana E. Norman, Scott B. Patten, Greg Freedman, Christopher J.L. Murray, Theo Vos, Harvey A. Whiteford

**Affiliations:** 1 University of Queensland, School of Population Health, Herston, Queensland, Australia; 2 Queensland Centre for Mental Health Research, Wacol, Queensland, Australia; 3 University of Queensland, Queensland Children's Medical Research Institute, Herston, Queensland, Australia; 4 University of Calgary, Department of Community Health Sciences, Calgary, Alberta, Canada; 5 University of Washington, Institute for Health Metrics and Evaluation, Seattle, Washington, United States of America; University of Western Sydney, Australia

## Abstract

**Background:**

Depressive disorders were a leading cause of burden in the Global Burden of Disease (GBD) 1990 and 2000 studies. Here, we analyze the burden of depressive disorders in GBD 2010 and present severity proportions, burden by country, region, age, sex, and year, as well as burden of depressive disorders as a risk factor for suicide and ischemic heart disease.

**Methods and Findings:**

Burden was calculated for major depressive disorder (MDD) and dysthymia. A systematic review of epidemiological data was conducted. The data were pooled using a Bayesian meta-regression. Disability weights from population survey data quantified the severity of health loss from depressive disorders. These weights were used to calculate years lived with disability (YLDs) and disability adjusted life years (DALYs). Separate DALYs were estimated for suicide and ischemic heart disease attributable to depressive disorders.

Depressive disorders were the second leading cause of YLDs in 2010. MDD accounted for 8.2% (5.9%–10.8%) of global YLDs and dysthymia for 1.4% (0.9%–2.0%). Depressive disorders were a leading cause of DALYs even though no mortality was attributed to them as the underlying cause. MDD accounted for 2.5% (1.9%–3.2%) of global DALYs and dysthymia for 0.5% (0.3%–0.6%). There was more regional variation in burden for MDD than for dysthymia; with higher estimates in females, and adults of working age. Whilst burden increased by 37.5% between 1990 and 2010, this was due to population growth and ageing. MDD explained 16 million suicide DALYs and almost 4 million ischemic heart disease DALYs. This attributable burden would increase the overall burden of depressive disorders from 3.0% (2.2%–3.8%) to 3.8% (3.0%–4.7%) of global DALYs.

**Conclusions:**

GBD 2010 identified depressive disorders as a leading cause of burden. MDD was also a contributor of burden allocated to suicide and ischemic heart disease. These findings emphasize the importance of including depressive disorders as a public-health priority and implementing cost-effective interventions to reduce its burden.

*Please see later in the article for the Editors' Summary*

## Abstract 2

In this paper, Ferrari and colleagues analyzed the burden of depressive disorders in GBD 2010 and identified depressive disorders as a leading cause of burden. The authors present severity proportions; burden by country, region, age, sex, and year; as well as burden of depressive disorders as a risk factor for suicide and ischemic heart disease.

*Please see later in the article for the Editors' Summary*

## Editors' Summary

**Background:**

Depressive disorders are common mental disorders that occur in people of all ages across all world regions. Depression—an overwhelming feeling of sadness and hopelessness that can last for months or years—can make people feel that life is no longer worth living. People affected by depression lose interest in the activities they used to enjoy and can also be affected by physical symptoms such as disturbed sleep. Major depressive disorder (MDD, also known as clinical depression) is an episodic disorder with a chronic (long-term) outcome and increased risk of death. It involves at least one major depressive episode in which the affected individual experiences a depressed mood almost all day, every day for at least 2 weeks. Dysthymia is a milder, chronic form of depression that lasts for at least 2 years. People with dysthymia are often described as constantly unhappy. Both these subtypes of depression (and others such as that experienced in bipolar disorder) can be treated with antidepressant drugs and with talking therapies.

**Why Was This Study Done?:**

Depressive disorders were a leading cause of disease burden in the 1990 and 2000 Global Burden of Disease (GBD) studies, collaborative scientific efforts that quantify the health loss attributable to diseases and injuries in terms of disability adjusted life years (DALYs; one DALY represents the loss of a healthy year of life). DALYs are calculated by adding together the years of life lived with a disability (YLD, a measure that includes a disability weight factor reflecting disease severity) and the years of life lost because of disorder-specific premature death. The GBD initiative aims to provide data that can be used to improve public-health policy. Thus, knowing that depressive disorders are a leading cause of disease burden worldwide has helped to prioritize depressive disorders in global public-health agendas. Here, the researchers analyze the burden of MDD and dysthymia in GBD 2010 by country, region, age, and sex, and calculate the burden of suicide and ischemic heart disease attributable to depressive disorders (depression is a risk factor for suicide and ischemic heart disease). GBD 2010 is broader in scope than previous GBD studies and quantifies the direct burden of 291 diseases and injuries and the burden attributable to 67 risk factors across 187 countries.

**What Did the Researchers Do and Find?:**

The researchers collected data on the prevalence, incidence, remission rates, and duration of MDD and dysthymia and on the excess deaths caused by these disorders from published articles. They pooled these data using a statistical method called Bayesian meta-regression and calculated YLDs for MDD and dysthymia using disability weights collected in population surveys. MDD accounted for 8.2% of global YLDs in 2010, making it the second leading cause of YLDs. Dysthymia accounted for 1.4% of global YLDs. MDD and dysthymia were also leading causes of DALYs, accounting for 2.5% and 0.5% of global DALYs, respectively. The regional variation in the burden was greater for MDD than for dysthymia, the burden of depressive disorders was higher in women than men, the largest proportion of YLDs from depressive disorders occurred among adults of working age, and the global burden of depressive disorders increased by 37.5% between 1990 and 2010 because of population growth and ageing. Finally, MDD explained an additional 16 million DALYs and 4 million DALYs when it was considered as a risk factor for suicide and ischemic heart disease, respectively. This “attributable” burden increased the overall burden of depressive disorders to 3.8% of global DALYs.

**What Do These Findings Mean?:**

These findings update and extend the information available from GBD 1990 and 2000 on the global burden of depressive disorders. They confirm that depressive disorders are a leading direct cause of the global disease burden and show that MDD also contributes to the burden allocated to suicide and ischemic heart disease. The estimates of the global burden of depressive disorders reported in GBD 2010 are likely to be more accurate than those in previous GBD studies but are limited by factors such as the sparseness of data on depressive disorders from developing countries and the validity of the disability weights used to calculate YLDs. Even so, these findings reinforce the importance of treating depressive disorders as a public-health priority and of implementing cost-effective interventions to reduce their ubiquitous burden.

**Additional Information:**

Please access these websites via the online version of this summary at http://dx.doi.org/10.1371/journal.pmed.1001547.

- The US National Institute of Mental Health provides information on all aspects of depression
- The UK National Health Service Choices website also provides detailed information about depression and includes personal stories about depression
- More personal stories about depression are available from healthtalkonline.org
- MedlinePlus provides links to other resources about depression (in English and Spanish)
- The World Health Organization provides information on depression and on the global burden of disease (in several languages)
- Information about the Global Burden of Disease initiative is available
- beyondblue provides many resources on depression
- The Queensland Centre for Mental Health Research provides information on epidemiology and the global burden of disease specifically for mental disorders

## Introduction

Depressive disorders are common mental disorders, occurring as early as 3 years of age and across all world regions [1]–[3]. Previous global burden of disease (GBD) studies in 1990 [4] and 2000 [5],[6] made notable contributions to shifting international focus towards depressive disorders as a leading cause of burden in its own right and also in comparison to more recognized physical disorders.

Using an approach first proposed in the World Development Report of 1993 [7], GBD 1990 and 2000 used disability adjusted life years (DALYs) to quantify the global burden attributable to diseases and injuries. One DALY represents the loss of a healthy year of life and aggregates the years of life lived with disability (YLD) with the years of life lost due to premature mortality (YLL) [4]–[6]. GBD 1990 ranked depressive disorders as the fourth leading cause of burden worldwide (equivalent to 3.7% of all DALYs) after lower respiratory infections, diarrhoeal diseases, and conditions arising during the perinatal period [4]. In GBD 2000, depressive disorders were the third leading cause of burden (equivalent to 4.3% of all DALYs) after lower respiratory infections and diarrhoeal diseases. It was also the leading cause of disability, responsible for 13.4% of YLDs in women and 8.3% in men [8].

These results have since made significant contributions to prioritising depressive disorders, and mental disorders as a group, in global public health agendas; particularly in promoting the addition of mental health interventions to health management plans [9]. For this purpose, it has also become important to provide comparable estimates of burden, reflective of recent statistical and epidemiological advancements in mental health research. This was a focus of the latest iteration of GBD (GBD 2010), which involved a substantial expansion of the GBD framework. GBD 2010 quantified the direct burden of 291 diseases and injuries, in parallel with the quantification of burden attributable to 67 risk factors. It included a complete epidemiological re-assessment of all diseases, injuries, and risk factors, across 187 countries, 21 world regions, males and females, 1990, 2005, 2010, and 20 different age groups. Unlike previous GBD studies, which estimated the burden of “unipolar depression” (i.e., a combination of the Diagnostic and Statistical Manual of Mental Disorders [DSM] [10] and the International Classification of Diseases [ICD] [11] categories [8],[12]), GBD 2010 quantified burden separately for major depressive disorder (MDD) and dysthymia; this was done to better accommodate differences in burden between the subtypes of depression. Rather than rely on a selective sample of data points (as was the case in previous GBD studies), burden estimation was based on a systematic review of the literature to obtain all available epidemiological data on MDD and dysthymia. Furthermore, revised estimation methods utilized modernized new statistical methods to model these epidemiological disease parameters, quantify disability, adjust for comorbidity between diseases, and propagate uncertainty into final burden estimates [13],[14].

This article follows the GBD 2010 capstone papers on the overarching methodology and findings of the study for all 291 diseases and injuries [13]–[18], and also the GBD 2010 mental and illicit drug use disorders research group's publication focusing on how mental and substance use disorders performed in comparison to other disease groups in GBD 2010 (see Figure S1 for an illustration of the GBD 2010 publications hierarchy) [19]. Here we focus on presenting the burden of MDD and dysthymia specifically. Analyzing burden estimates at the national, regional, and individual characteristic level is important from both a clinical and population-health perspective to identify populations most at risk. We summarise the updated methodology and inputs used for the computation of YLDs, YLLs, and DALYs and present an analysis of country-, region-, age-, sex-, and, year-specific trends in the burden of depressive disorders. We also address a criticism of previous GBD studies [9] by estimating the additional burden attributable to MDD as a risk factor for other health outcomes.

## Methods

### Case Definition

The DSM-IV-TR [10] describes MDD (296.21–24, 296.31–34), as an episodic disorder with a chronic outcome and an elevated risk of mortality, equivalent to ICD-10's description of recurrent depressive disorder (F32.0–9, F33.0–9) [11]. It involves the presence of at least one major depressive episode, which is the experience of depressed mood almost all day, every day, for at least 2 weeks. As dysthymia (DSM-IV-TR: 300.4; ICD-10: F34.1) involves a less severely depressed mood compared to MDD and a duration of at least 2 years, it is described as chronic rather than episodic, with low rates of remission and no elevated risk of mortality [10],[11].

### Calculation of Direct Burden-YLDs

The estimation of YLDs for a given disorder can be understood as a synthesis of epidemiological data that not only accommodates the number of people affected but also the severity and disability associated with their symptoms [18]. In GBD 2010, prevalent rather than incident YLDs were calculated, without age-weighting and discounting [13]. This means that for GBD 2010, YLDs were calculated by multiplying the prevalence of a given disorder by its corresponding severity- and comorbidity-adjusted disability weight. As these choices fundamentally change the metric, YLDs for 1990 were re-estimated using the same methods to allow meaningful comparisons of changes over time.

#### Epidemiological inputs

For MDD and dysthymia, prevalence, incidence, remission or duration, and excess mortality data were captured through a systematic review of the literature between 1st January 1980 and 31st December 2008 and continued perusal of the literature until 31st December 2011. A search of relevant online databases (Medline, PsycInfo, and EMBASE) was conducted as per the Preferred Reporting Items for Systematic Reviews and Meta-Analyses (PRISMA) [20]. To be eligible for inclusion studies needed to report estimates: of prevalence, incidence, duration, and/or excess mortality from 1980 onwards; representative of the community, region, or country under investigation; and based on DSM or ICD definitions of MDD and dysthymia. For prevalence, we required point (current/past month) or past year prevalence estimates. Lifetime estimates were excluded as recall bias invalidates them as credible measures of disease burden [21]–[24]. For incidence, we used hazard rates with person years of follow-up as the denominator. Given the episodic presentation of MDD, we used data on the duration of major depressive episodes from follow-up studies of the natural history of the disorder. For dysthymia we used remission data from follow-up studies capturing cases no longer fulfilling diagnostic criteria for the disorder. For excess-mortality, we used estimates of relative-risk (RR) or a standardised mortality ratio. Information on this systematic review can be accessed in previous publications [1],[3],[25],[26], with the main findings highlighted in Tables 1 and S1.

**Table 1:** Summary of data used to calculate YLDs for depressive disorders.

Parameter	MDD	Dysthymia	Source
**Epidemiological inputs**			Systematic review of the literature [1],[3].
** Number of data points (and studies)**			
Prevalence	544 (116)	141 (36)	
Incidence	19 (4)^a^	3 (2)^a^	
Remission	—	3 (2)	
Duration	1 (5)^b^	—	
Excess-mortality	14 (11)	5 (2)^c^	
** DisMod-MR point prevalence % (95% UI) and cases**			DisMod-MR epidemiological modelling [2],[3]
Global prevalence	4.4% (4.1%–4.7%); 298 million cases	1.55% (1.5%–1.6%); 106 million cases	
Males	3.2% (3.0%–3.6%); 111 million cases	1.3% (1.2%–1.4%); 44 million cases	
Females	5.5% (5.0%–6.0%);187 million cases	1.8% (1.7%–1.9%); 62 million cases	
**Disability weights**			Derived by GBD core group and mental disorders expert group for the GBD 2010 disability weight survey [17].
**Health state lay descriptions**			
Mild	Has constant sadness and has lost interest in usual activities. The person can still function in daily life with extra effort, but sleeps badly, feels tired, and has trouble concentrating	—	
Moderate	Has constant sadness and has lost interest in usual activities. The person has some difficulty in daily life, sleeps badly, has trouble concentrating, and sometimes thinks about harming himself (or herself).	—	
Severe	Has overwhelming, constant sadness and cannot function in daily life. The person sometimes loses touch with reality and wants to harm or kill himself (or herself)	—	
**Raw disability weights (95% UI)**			GBD 2010 disability weight Survey [17].
Mild	0.16 (0.11–0.22)	0.16 (0.11–0.22)^d^	
Moderate	0.41 (0.28–0.55)		
Severe	0.66 (0.47–0.82)		
**Severity distribution %(95% UI)**			Based on SF–12 data from MEPS, NSMHWB, and NESARC [18].
Asymptomatic	13.9% (10.2%–17.7%)	29.2% (24.9%–33.6%)	
Mild MDD/Symptomatic dysthymia	58.8% (48.0%–68.5%)	70.8% (66.4%–75.1%)	
Moderate	16.5% (12.1%–21.0%)		
Severe	10.8% (3.8%–20.3%)		
**Average disability weight (95% UI)**	0.23 (0.18–0.30)	0.11 (0.07–0.15)	Based on severity proportions from MEPS, NSMHWB, and NESARC data, applied to weights from GBD 2010 disability weights survey [18].

a Incidence data were excluded for MDD and dysthymia as they were not consistent with the prevalence and duration/remission data.

b The one data point for duration of 37.7 weeks was an estimate of average duration calculated from a best fit curve between the data points available from five studies.

c Both studies reported no elevated risk of mortality in those with dysthymia.

d The disability weight for mild-MDD was applied to dysthymia.

95% UI, 95% uncertainty interval.

#### Disease modelling

For each disorder, epidemiological estimates from the literature review were pooled using DisMod-MR, a Bayesian meta-regression tool developed specifically for GBD 2010 [18]. DisMod-MR is based on a generalized negative binomial model that: (1) uses an Incidence-Prevalence-Mortality mathematical model [18],[27] to enforce internal consistency between estimates from different epidemiological parameters; (2) estimates data for countries and world regions with no or few available input data based on random effects for country, regions, and their corresponding super-region groupings; (3) deals with variability in the data due to measurement bias or alternatively ecological factors through the use of study- and country-level covariates; and (4) propagates uncertainty around the raw epidemiological data through to the final estimates [18]. The DisMod-MR output required for YLD estimations were prevalence estimates (including their respective 95% uncertainty intervals) for 187 countries, 21 world regions, males and females, 1990, 2005, and 2010, for 20 age groups. The global point prevalence output has been summarised in Table 1 and the country-level output in Table S2. Given that the focus of this article was to report on the burden of depressive disorders, we have only summarised the disease modelling process here. More details on the disorder-specific modelling methodology, output, and, sensitivity analyses around final estimates have been reported in separate publications [2],[3].

#### Disability weights

The GBD 2010 framework describes disability as any short-term or long-term loss of health associated with a given health state [17]. Unlike GBD 1990, which estimated disability weights by expert deliberation [4], GBD 2010 captured community-representative data through population surveys in Bangladesh, Indonesia, Peru, Tanzania, and the United States of America (14,710 participants) and an open-access internet survey available in English, Spanish, and Mandarin (16,328 participants). Each survey included lay descriptions of 220 health states, which together parsimoniously described the non-fatal consequences of all diseases and injuries in GBD 2010. These were presented as paired-comparison questions asking participants to decide which of two randomly selected health states they considered the healthier. Responses were anchored on a scale of 0 (healthy) to 1 (death) with some additional “population health equivalence” questions, which compared the overall health benefits of different life saving or disease prevention programs, to derive disability weights [17].

#### Severity distribution

In order to capture the range of severity in the presentation of MDD, disability weights were estimated for mild, moderate, and severe states of MDD. The choice of health states and their lay descriptions (Table 1) were formulated by members of the GBD mental disorders expert group, under the guidance of the GBD core group. The aim here was to encapsulate the main features of MDD and dysthymia (as described by DSM-IV and ICD-10 [10],[11]), using consistent, brief, and clear wording across each health state. Given the milder and more stable presentation of dysthymia, it was allocated the same disability weight as that for mild MDD.

Information on the distribution of mild, moderate, and severe cases of MDD was obtained from the US Medical Expenditure Panel Survey (MEPS) 2000–2009 [28], the US National Epidemiological Survey on Alcohol and Related Conditions (NESARC) 2000–2001 and 2004–2005 [29], and the Australian National Survey of Mental Health and Wellbeing of Adults (NSMHWB) 1997 [30]; these surveys captured the prevalence of multiple mental and physical disorders included in GBD 2010 (156 in MEPS; 32 in NESARC; 20 in NSMHWB) as well as health status information measured by the Short Form 12-item (SF-12) [31].

A crosswalk between a score on the SF-12 and the GBD 2010 disability weights was derived from a convenience sample of participants asked to fill in the SF-12 to reflect 62 lay descriptions of health states of varying severity. From a mathematical relationship between SF-12 summary scores and disability weights, SF-12 values were translated into disability weights for all respondents in the MEPS, NESARC, and NSMHWB reflecting the combined severity of any comorbid condition. Next, a regression with random effects for all comorbid health states was run to parse disability in each individual to each comorbid health state [18]. Once disability attributable to comorbid disorders was portioned out, 14% of MDD cases and 29% of dysthymia cases had no disability (i.e., a disability weight of 0) at the time of the survey. Cases scoring a disability weight of >0 counted as symptomatic. For MDD, symptomatic cases were further disaggregated into mild, moderate, and severe where cases scoring a disability weight of >0 to halfway between a corresponding score of mild and moderate on the SF-12 counted as mild; cases scoring from there to halfway between a corresponding SF-12 score of moderate and severe counted as moderate; and those scoring from there onwards counted as severe. The proportion of cases in each state was then multiplied by its disability weight and summed to obtain an overall disability weight for MDD. Overall, the proportion of cases in asymptomatic, mild, moderate, and severe states over the course of MDD was almost identical across MEPS, NESARC, and NSMHWB for 12-month prevalence. As the NSMHWB was the only survey with one-month diagnoses and the SF-12 questions pertain to severity in the past month we used the distribution of severity from the NSMHWB for one-month diagnoses. Table 1 summarises the resulting health state proportions and disability weights. More details on this methodology have also been provided elsewhere [18].

#### Comorbidity adjustment

GBD 2010 YLD estimates were adjusted for the effect of comorbidity between diseases. Hypothetical populations by age, sex, year, and country were estimated using microsimulation. For each individual in the hypothetical population: (1) prevalence data for all GBD sequelae were used to estimate the probability of experiencing no, one, or more than one disabling condition (i.e., health state); (2) from this, a combined disability weight capturing disability attributable to each comorbid condition was estimated with a multiplicative function and; (3) re-distributed to individual conditions in a manner that was proportional to the disability weight of each condition in isolation; (4) the decrease between the original disability weights for MDD and dysthymia and the adjusted disability weights was considered as the “comorbidity correction” for YLDs. As we were unable to find sufficiently large datasets to explore and quantify the difference in disability due to comorbidities that were dependent versus independent of each other, only the latter was taken into consideration here. In support for this step, the severity adjustments using MEPS data showed that estimating independent comorbidity (i.e., assuming no correlation between comorbid conditions), using a multiplicative approach, explained most of the modulating effect of comorbidity on disability. The GBD 2010 approach to comorbidity has been discussed in greater detail elsewhere [18].

#### Time trend analysis

We replicated the methodology presented in the GBD 2010 capstone YLD paper [18] to disaggregate the change in YLDs between 1990 and 2010 into changes due to population growth, population age and sex structure, and YLD rates (i.e., the disorder's epidemiology). This process involved estimating the total YLDs anticipated in 2010 if: (1) population growth increased to 2010 levels but the population age/sex structure and YLD rates remained the same as in 1990; and (2) the age/sex-population structure was at 2010 levels but the YLD rates remained the same as in 1990.

### Calculation of Direct Burden-DALYs

We calculated DALYs as the sum of YLDs and YLLs. YLLs are calculated by multiplying the number of deaths due to the given disorder at a particular age by the standard life expectancy at that age. However, death records used in GBD 2010 followed ICD-10 rules for categorical attribution of cause of death to a single underlying cause [11] and, therefore, did not document any deaths due to depressive disorders. As such, we were unable to calculate disorder-specific YLLs for depressive disorders. Instead, associated deaths were captured under other causes in the GBD cause list and needed to be re-attributed to depressive disorders.

### Calculation of Attributable Burden

The comparative risk assessment (CRA) component of GBD 2010 quantified the burden attributable to each risk factor exposure compared to an alternative (counterfactual) exposure distribution [15]. Diseases, like MDD, can also be considered risk factors for loss of health if associated with elevated risk of mortality or disability from other diseases or injuries. We replicated the GBD 2010 CRA methodology to investigate the additional burden attributable to depressive disorders as a risk factor for other health outcomes. The burden of disease attributable to depressive disorders was estimated by comparing the current health status with a theoretical-minimum-risk exposure distribution, the optimum exposure distribution with the lowest possible risk. For depressive disorders the theoretical minimum was defined by the counterfactual status of absence of the disease. This process involved (1) the selection of health outcomes attributable to MDD and dysthymia based on data availability and adherence to criteria about causality; (2) conducting systematic reviews of the literature and meta-analyses of effect sizes of the disorder-outcome pairing (the gold standard for effect measure were RR estimates by year and sex derived from prospective cohort studies with a naturalistic follow-up of cases, representative of the general population); (3) combining the pooled RR estimates with the DisMod-MR prevalence output for the disorder to calculate population attributable fractions (PAFs); and (4) multiplying PAFs by the corresponding cause-specific DALYs for the outcome under investigation to calculate attributable burden. The process allowed us to estimate attributable burden by sex, age, year, region, and country. Out of the comprehensive list of health outcomes originally investigated for mental disorders [32], there was sufficient evidence for causal effects to quantify the burden attributable to MDD as a risk factor for suicide and ischemic heart disease. These literature searches have been reported in greater detail elsewhere [33],[34] with the main results highlighted in Table 2.

**Table 2:** Summary of data used to calculate burden attributable to MDD as a risk factor for suicide and ischemic heart disease.

Outcome	Suicide	Ischemic Heart Disease
**Number of data points (and studies)**	4 (3)	13 (8)
**Number countries**	2	2
**Pooled RR (95% UI)** a	19.9 (9.5–41.7)	1.6 (1.3–1.9)

a RR estimates were pooled using meta-analytic strategies [33],[34].

95% UI, 95% uncertainty interval;

Where we report comparisons of prevalence, YLDs, or DALYs by country or region we use ISO 3166-1 alpha 3 codes (http://www.iso.org/iso/home/standards/country_codes.htm) and age-standardised values using direct standardisation to the global standard population proposed by the World Health Organization in 2001 (http://www.who.int/healthinfo/paper31.pdf).

## Results

### Direct Burden of Depressive Disorders

Out of a total of 2.5 billion DALYs generated in the year 2010, mental and substance use disorders accounted for 7.4% (95% uncertainty interval: 6.3%–8.6%), depressive disorders for 3.0% (2.2%–3.8%), MDD for 2.5% (1.9%–3.2%), and dysthymia for 0.5% (0.3%–0.6%). MDD ranked as the 11th and dysthymia as the 51st leading cause of global DALYs in 2010. DALYs for both MDD and dysthymia were based solely on YLDs as there were no disorder-specific deaths (and therefore YLLs) recorded for either disorder. MDD was the second leading cause explaining 8.2% (5.9%–10.8%) of all YLDs, after low back pain. Dysthymia ranked as the 19th leading cause, explaining 1.4% (0.9%–2.0%) of all YLDs in 2010.

Although the global YLD rankings were the same in 1990, depressive disorders caused only 9.3% (6.7%–12.2%) of all YLDs, corresponding with a 37.5% increase in YLDs between 1990 and 2010 (see Table 3). The increase was entirely accounted for by population growth and ageing with no substantial change in age-specific prevalence.

**Table 3:** Change in depressive disorder YLDs between 1990 and 2010.

Total YLDs in 1990 and 2010	MDD	Dysthymia	Depressive Disorders
Total YLDs in 1990	46,138,600	7,870,700	54,009,300
Total YLDs in 2010	63,179,247	11,084,100	74,261,500
Total YLDs generated from 2010 population, 1990 population age structure, 1990 YLD rates (step 1)	59,904,870	10,067,939	69,972,809
Total YLDs generated from 2010 population, 2010 population age structure, 1990 YLD rates (step 2)	64,537,300	11,061,231	75,598,531
Total change in YLDs between 1990 and 2010	36.9%	40.8%	37.5%
Change in YLDs between 1990 and 2010 due to population growth	29.8%	27.9%	29.6%
Change in YLDs between 1990 and 2010 due to population aging	10.0%	12.6%	10.4%
Change in YLDs between 1990 and 2010 due to prevalence increase	−2.9%	0.3%	−2.5%

The difference between total YLDs in 1990 and YLDs at step 1 represents the change in YLDs due to population growth; the difference between YLDs at step 1 and YLDs at step 2 represents the change in YLDs due to population aging; the difference between total YLDs in 2010 and YLDs at step 2 represents the change in YLDs due to changes in prevalence.

**Figure 1:**
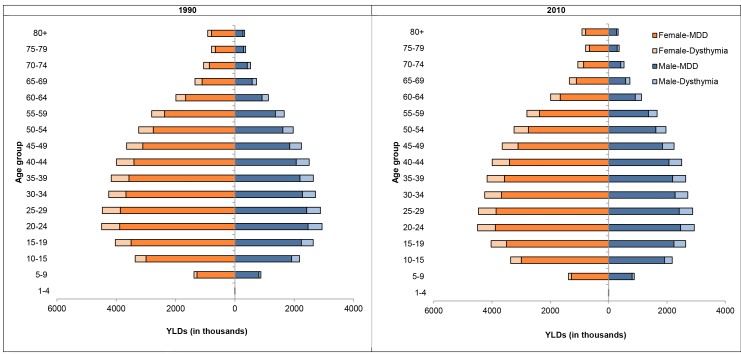
YLDs by age and sex for MDD and dysthymia in 1990 and 2010.

Figure 1 shows the composition of YLDs by age and sex for MDD and dysthymia in 1990 and 2010. YLDs were consistently higher for MDD compared to dysthymia and also in females compared to males. There were changes across the lifespan with YLDs peaking in the twenties and gradually decreasing into the older ages. Globally in 2010, the largest proportion of YLDs from depressive disorders occurred at working ages (15 to 64 years) with 60.4 million YLDs, followed by the 0 to 14 year age group with 7.8 million YLDs, and the 65 and over age group with 6.1 million YLDs.

**Figure 2:**
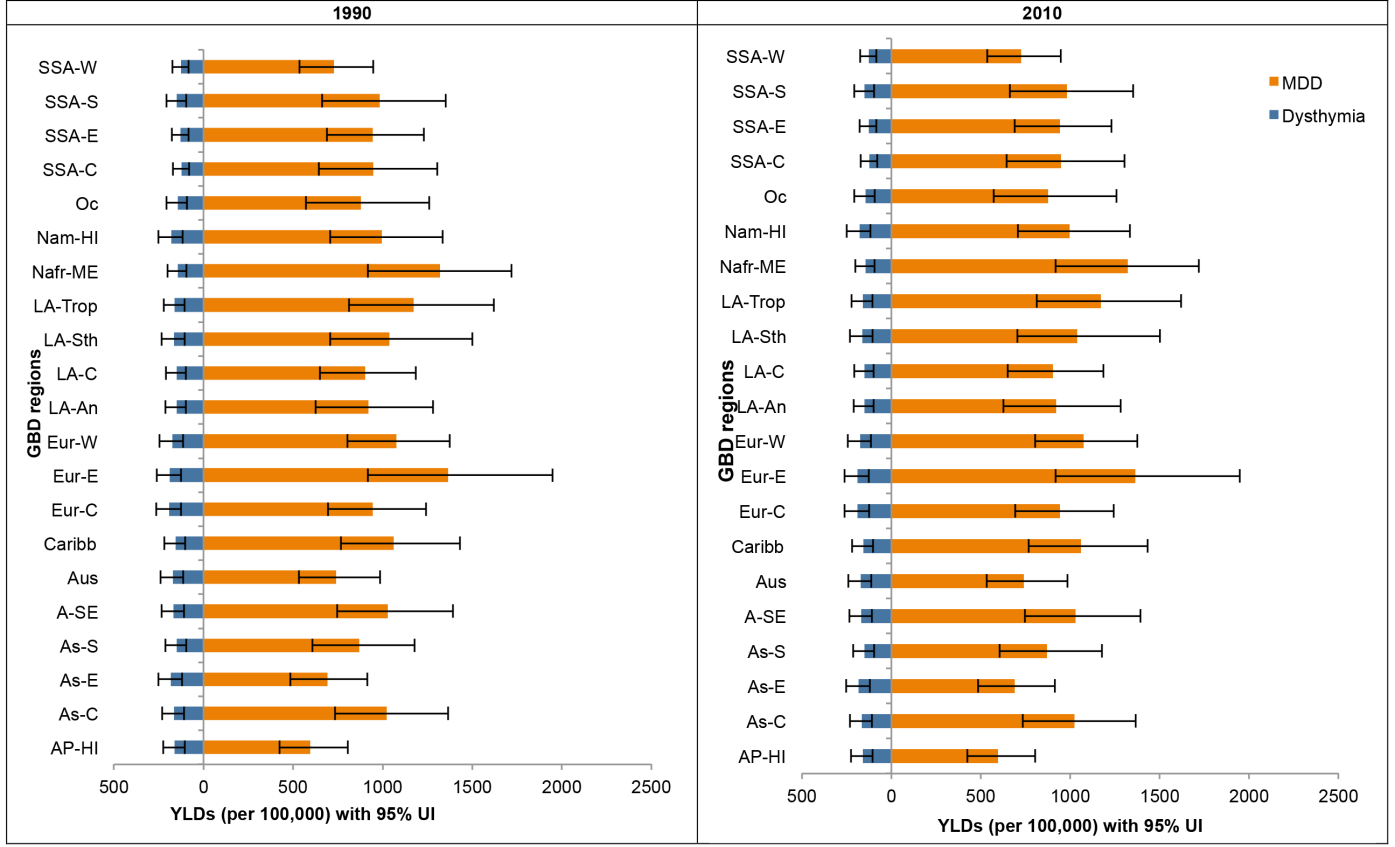
YLD rates (per 100,000) by region for MDD and dysthymia in 1990 and 2010. 95% UI, 95% uncertainty interval; AP-HI, Asia Pacific, high income; As-C, Asia Central; AS-E, Asia East; AS-S, Asia South; A-SE, Asia Southeast; Aus, Australasia; Caribb, Caribbean; Eur-C, Europe Central; Eur-E, Europe Eastern; Eur-W, Europe Western; LA-An, Latin America, Andean; LA-C, Latin America, Central; LA-Sth, Latin America, Southern; LA-Trop, Latin America, Tropical; Nafr-ME, North Africa/Middle East; Nam-HI, North America, high income; Oc, Oceania; SSA-C, Sub-Saharan Africa, Central; SSA-E, Sub-Saharan Africa, East; SSA-S, Sub-Saharan Africa Southern; SSA-W, Sub-Saharan Africa, West.

**Figure 3:**
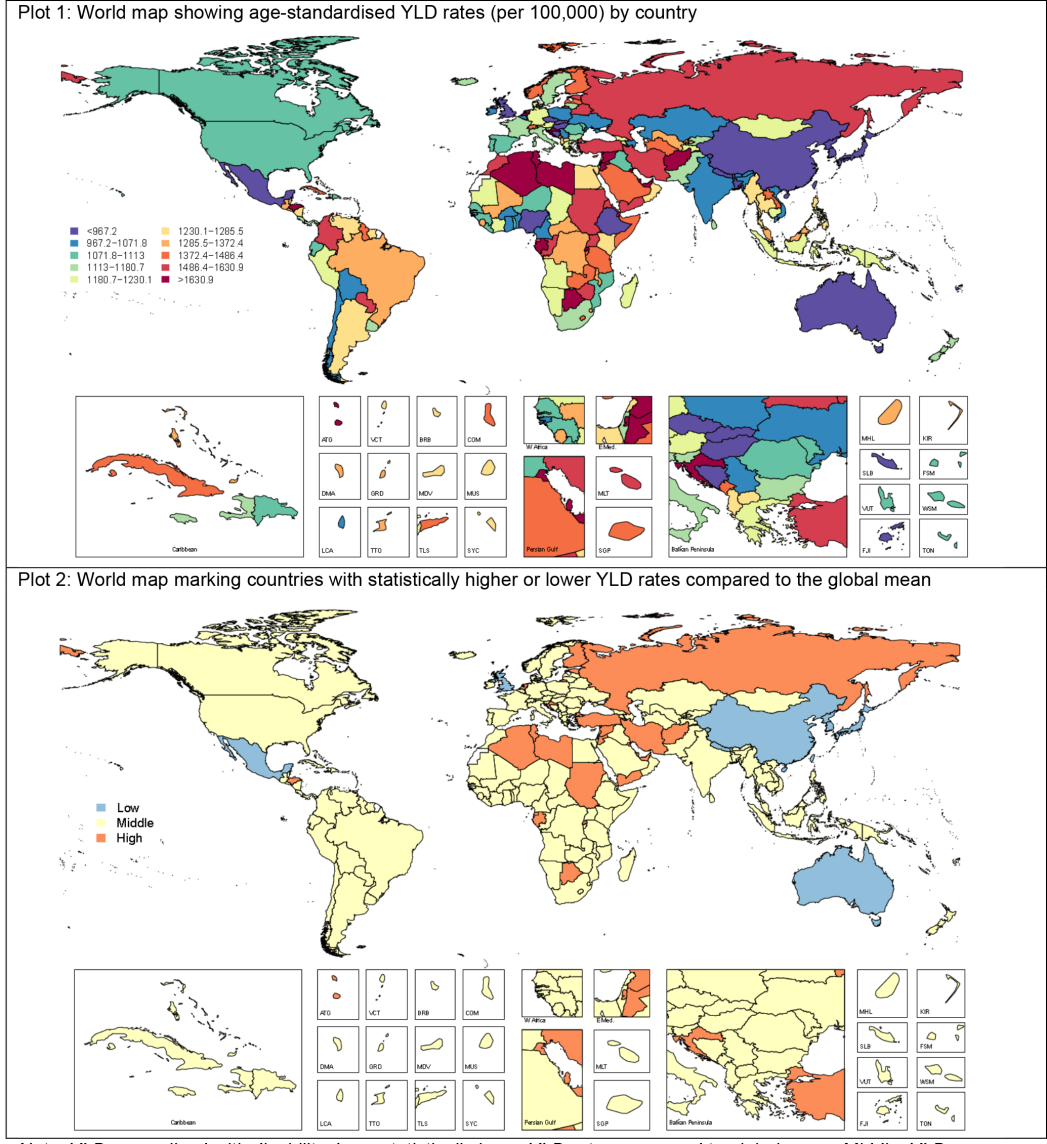
YLD rates (per 100,000) by country for depressive disorders in 2010. Low, statistically lower YLD rates compared to global mean; middle, YLD rates not statistically different to global mean; high, statistically higher YLD rates compared to global mean.

Figure 2 shows the composition of YLD rates by region for MDD and dysthymia in 1990 and 2010. Although dysthymia YLD rates were consistent between regions, there were differences for MDD. While the focus of GBD 2010 publications so far has largely been on reporting regional and global burden estimates, all analyses were primarily conducted at the country level. On the basis of these country-level analyses, Figure 3 shows the composition of YLD rates in 2010 (with the corresponding 1990 estimates presented in Figure S2) by country for MDD and dysthymia combined (plot 1) and countries with statistically higher or lower YLD rates than the global mean (plot 2); the latter of which also needs to be considered while interpreting country-level findings. Most of the regional, and country-level differences in YLDs, were within wide and overlapping ranges of uncertainty, with only a few countries with statistically higher or lower YLD rates compared to the global mean. YLD rates were highest in Afghanistan (included in North Africa/Middle East) and lowest in Japan (included in the Asia Pacific, high income).

Table 4 summarises the regional YLD and DALY rankings for MDD and dysthymia in 2010 (with the corresponding 1990 rankings presented in Table S3). This information highlights how MDD and dysthymia ranked in burden in comparison to other diseases and injuries in GBD 2010. MDD ranked as the 11th leading cause of DALYs globally but was as high as third in North Africa/Middle East and Latin America, Andean, and as low as 19th in sub-Saharan Africa, West. Although these regional rankings differed substantially to their corresponding global ranking, the overlapping 95% uncertainty intervals around some mean ranks also need to be considered.

**Table 4:** Regional DALY and YLD rankings with 95% uncertainty intervals for depressive disorders in 2010.

Region	YLDs				DALYs			
	MDD		Dysthymia		MDD		Dysthymia	
	Order	Mean Rank (95% UI)	Order	Mean Rank (95% UI)	Order	Mean Rank (95% UI)	Order	Mean Rank (95% UI)
**Global**	**2**	**1.9 (1–3)**	**19**	**18.6 (13–26)**	**11**	**10.8 (7–14)**	**51**	**51.2 (42–62.5)**
Asia Pacific, high income	4	4.3 (2–7)	22	21.1 (14–28)	12	11.5 (6–17)	35	35.9 (27–47)
Asia Central	1	1.5 (1–3)	19	19.4 (14–26)	6	7.2 (4–12)	46	46.7 (38–56)
Asia East	2	2.3 (1–3)	16	15.1 (9–21)	8	8.4 (5–12)	33	32.4 (22–42.5)
Asia South	3	2.9 (1–4)	20	19.8 (11–29)	14	13.3 (8–18)	55	54.7 (41–70)
Asia Southeast	1	1.4 (1–2)	19	17.9 (10–26)	6	6.7 (3–11)	44	45.1 (36–57)
Australasia	2	2.9 (2–7)	21	20.8 (14–28)	4	6.1 (3–14)	33	34.5 (23–47)
Caribbean	2	2.3 (1–4)	22	23 (18–33)	7	8.6 (4–13)	52	52.1 (41–65)
Europe Central	2	2.2 (2–4)	20	19.2 (13–26)	5	6.6 (4–10)	36	37.4 (28–52)
Europe Eastern	2	1.8 (1–2)	20	19.3 (14–26)	5	5.6 (3–9.5)	43	45.2 (35–59.5)
Europe Western	2	2.1 (2–3)	20	20.7 (15–28)	4	4.2 (3–8)	36	36.7 (27–51)
Latin America, Andean	1	1.7 (1–3)	22	20.9 (15–28)	3	4.6 (2–10.5)	42	43.5 (35–57)
Latin America, Central	1	1.3 (1–2)	19	19.1 (13–26)	5	5.2 (3–10)	41	40.1 (31–52)
Latin America, Southern	2	1.6 (1–3)	20	20.2 (13–28)	4	3.4 (2–6.5)	41	42.0 (32–58)
Latin America, Tropical	2	1.8 (1–2)	20	20.2 (14.5–27)	6	5 (2.5–7)	42	42.8 (35–53)
North Africa/Middle East	2	1.9 (1–2)	19	19.6 (15–28)	3	3.8 (2–8)	44	42.9 (32.5–55)
North America, high income	2	2.1 (1–4)	21	20.2 (14–27)	5	5.0 (2–10)	38	38.1 (30–50)
Oceania	1	1.6 (1–4)	23	22.4 (15–32)	12	13.4 (6–23.5)	65	63.1 (51–75)
Sub-Saharan Africa, Central	2	2.0 (1–3)	31	28.0 (18–37)	17	17.9 (12–24)	64	61.8 (50–75)
Sub-Saharan Africa, East	2	2.0 (1–3)	20	22.5 (14–35)	13	14.2 (11–18)	54	55.5 (43–75)
Sub-Saharan Africa Southern	2	2.5 (1–5)	22	22.6 (14–32)	10	10.4 (6–16)	52	52.3 (43–64)
Sub-Saharan Africa, West	3	3.1 (2–4)	27	26.1 (18–34)	19	19.7 (14–26)	58	58.4 (46–72)

Mean rank, YLD, and DALY ranks were estimated for MDD and dysthymia then simulated 1,000 times to estimate 95% uncertainty ranges. The 95% bounds of uncertainty represent the 25th and 975th value of the 1,000 draws; order, regional YLDs, and DALYs for MDD and dysthymia were ordered by their mean rank across 1,000 draws.

95% UI, 95% uncertainty interval.

### Attributable Burden

The above estimates reflect direct disability where MDD is selected as the underlying cause but exclude the excess deaths resulting from the increased risk of mortality from suicide and burden from ischemic heart disease attributed to MDD as a risk factor. In 2010, MDD explained a further 16 million DALYs when it was considered as a risk factor for suicide. Overall, close to half (46.1% [28.03%–60.8%]) of DALYs originally allocated to suicide (included as intentional injuries in the GBD cause list) could be re-attributed to MDD. In addition to this, 2.9% (1.5%–4.5%) of ischemic heart disease DALYs (3.8 million DALYs of which 93.5% were YLLs) was attributable to MDD. Adding these to MDD would have increased the overall burden of MDD from 2.5% (1.9%–3.2%) to 3.4% (2.7%–4.2%) of global DALYs and the overall burden of depressive disorders from 3.0% (2.2%–3.8%) to 3.8% (3.0%–4.7%) of global DALYs. The global burden rankings of MDD in the GBD cause list would have increased from eleventh to eighth place, surpassing road injury, chronic obstructive pulmonary disease, and preterm birth complications.

## Discussion

GBD 2010 has identified depressive disorders as one of the leading causes of YLDs. In spite of the lack of disorder-specific YLLs, it was also a leading cause of DALYs, emphasizing the importance of non-fatal health outcomes in the quantification of disease burden. Within depressive disorders, MDD was the main contributor to burden, accounting for 85% of YLDs and DALYs in 2010. This finding was driven by high prevalence estimates with 298 million MDD cases in 2010 [2] and 106 million cases of dysthymia [3]. Discounting and age-weighting in previous GBD studies contributed in part to the high ranking of mental disorders. Despite not discounting (and therefore giving greater weight to mortality than disability) and not age-weighting (and therefore giving less weight to disabling conditions in young and middle aged adults) depressive disorders are still a leading cause of disability.

GBD 2010 quantified burden for 1990, 2005, and 2010 allowing comparisons of estimates over time based on comparable methods. Contrary to recent literature on the topic [35],[36], our findings suggest that the epidemiology of both MDD and dysthymia remained relatively stable over time. There was a slight decrease in the prevalence rate of MDD between 1990 and 2010 but this was too small to allow for any explicit interpretation. As noted earlier there was a 37.5% increase in YLDs between 1990 and 2010 due to population growth and ageing. This has important implications for global health, especially in developing countries where increased life expectancy due to better reproductive health, nutrition, and control of childhood infectious diseases means more of the population are living to the age where depressive disorders are prevalent.

Our findings not only emphasize depressive disorders as a global health priority, but also highlight the importance of understanding the variations both between and within regions when setting global health objectives. Variations in burden rankings between regions can be masked while considering global-level findings. For instance, some regional DALY rankings of MDD and dysthymia were considerably different than their corresponding global ranking. In the case of North Africa/Middle East, conflict in the region increased the prevalence of MDD, leading to a higher burden ranking for MDD. In sub-Saharan Africa on the other hand, the larger burden of communicable diseases such as malaria and HIV/AIDs resulted in a relatively lower ranking of MDD and dysthymia [14].

GBD 2010's capacity to generate country-level burden as well as regional estimates was especially relevant for MDD, which has been linked to risk factors such as conflict [2],[37], intimate partner violence, and child sexual abuse [15], the levels of which vary between countries. Nevertheless, it's important to stress that variation (or in some cases lack of variation) in burden estimates and rankings may reflect the true distribution of burden, a lack of available epidemiological data, or outliers that can occur by chance in any distribution. The nature of the DisMod-MR modelling strategy used was such that if raw epidemiological data were not available for a given country, prevalence estimates were imputed on the basis of random effects for country, region, and super-region and fixed effects for country-level covariates such as the mortality rate due to conflict. In the case of MDD, as previously stated, our literature review was able to capture prevalence data from conflict countries such as Afghanistan and Iraq. To improve the predictive power of our DisMod-MR model, we included conflict and post conflict status covariates to guide the DisMod-MR estimation of MDD prevalence for regions with no data [2]. This strategy does not replace high quality primary data but we preferred computing burden estimates for these countries/regions rather than excluding them entirely from this global health exercise. The global availability of the raw epidemiological data for MDD and dysthymia has been summarised in Table S1 as well as in previous publications [1]–[3]. Any utilization of GBD country-level estimates will have to take these data into consideration [38]–[40]. As the updating of GBD continues we hope the scrutiny of these country-level findings will promote primary data collection on the epidemiology of depressive disorders, particularly in developing countries where data are sparse.

We found no evidence of deaths attributable to dysthymia; this was consistent with our investigations into the epidemiology of dysthymia, finding no excess mortality attributable to the disorder [3]. We found evidence for an elevated risk for mortality in those diagnosed with MDD [2],[25]; however, since a health outcome could only occur once in the GBD cause list, MDD related deaths from suicide and ischemic heart disease were captured under the headings of intentional injuries and cardiovascular disease in the GBD capstone papers [14],[18]. In this article, we've attributed a fraction of these DALYs to MDD using counterfactual estimation and GBD 2010 CRA methodology [15]. The addition of these outcomes would have shifted MDD from eleventh to eighth leading cause of DALYs, further supporting the prioritisation of depressive disorders in the prevention and management of wider aspects of health.

It is worth noting that we were unable to quantify burden for all the outcomes of MDD and dysthymia. As a result, it is likely that the burden estimates presented here still underestimate the true burden of depressive disorders. Although there is literature linking stroke, diabetes, and vascular dementia/Alzheimer's disease to MDD, there was insufficient evidence at the time of our review for a causal relationship and more studies are needed to support these tentative associations [32]. For instance, many studies relied on symptom scales rather than DSM/ICD criteria to capture people with MDD and are hence likely to overestimate the strength of these associations. As more rigorous evidence is made available we aim to quantify the burden due to MDD as a risk factor of other causes. Furthermore, for both suicide and ischemic heart disease, meta-analyses relied on data from two countries that met our inclusion criteria. There is also uncertainty as to the extent to which these effect sizes are generalizable to different populations and GBD regions; this too is an area for further research.

New to GBD 2010 was the capability of propagating uncertainty from the epidemiological data points through to burden estimates. While this also included uncertainty introduced by the adjustment of data points for study quality variables, the true uncertainty may be larger yet as we did not account for the rather crude nature of the study quality covariates as binary variables applied equally at all ages and both genders. The aim of GBD 2010 was to provide an empirical platform for consistently comparing the global burden attributable to different diseases and injuries. Given that MDD and dysthymia represented only two out of 291 causes included in the study, it was not surprising that some elements of the burden methodology could not be completely tailored to them. With ongoing improvements to the GBD methodology and the growing availability of epidemiological data, we will be able to add to our understanding of the global burden of depressive disorders.

It is also worth acknowledging that our findings were reliant on the validity of the disability weights used. Although the methodology used to quantify disability largely improved on what was used in GBD 1990, some areas could benefit from further refinement. The health state definitions and subsequent lay descriptions for MDD and dysthymia may not have been representative of all participants' experiences of the disorder. Further research is required into whether different health state definitions would change disability weights and, ultimately, burden estimates. Analyses of the disability weight surveys suggested a high degree of consistency between disability weights from the country surveys and the internet survey. In spite of responses coming from a heterogeneous sample of individuals (e.g., a high proportion of highly educated individuals from the internet-based survey and the opposite from the population-based survey from Tanzania), the strength of the correlation between disability weights was at least 0·9 across all surveys except in Bangladesh where it was 0·75 [17]. That said, although these high correlations lend support to the argument that the disability weights used can be generalized across countries, replication of the disability weights survey in other settings is required for clearer conclusions.

Our review of the literature also indicated that there was much less reported on the severity of MDD and dysthymia compared to other areas of the disorders' epidemiology. Moreover, the available literature differed vastly in sampling methods and survey instruments hence capturing different conceptualisations of severity with no general consensus in distinguishing between mild, moderate, and severe states of MDD [41]. For instance, severity distributions obtained from the World Mental Health Survey study group indicated the majority of cases with MDD were classified as severe. The skew towards classifying cases as severe was partly due to the algorithm used to group answers to questions from the Sheehan Disability Scale and/or the Quick Inventory of Depressive Symptomatology [42]–[44] and partly due to the inclusion of additional criteria related to comorbid health states rendering the classification as unusable for GBD purposes [42],[45]. So instead, we turned to data from the MEPS, NESARC, and NSMHWB, which provided a less skewed distribution of cases and allowed us to derive severity distributions while also controlling for comorbidity. However, these three surveys were from two high income countries, limiting the global representativeness of our severity distributions and making it impossible to quantify any effect of treatment on severity. There is a clear need for further investigations with comparable methods into the severity distribution of MDD and dysthymia and the variation thereof between countries and by levels of access to care.

### Conclusions

Our findings not only highlight the fact that depressive disorders are a global health priority but also that it is important to understand variations in burden by disorder, country, region, age, sex, and year when setting global health objectives. Furthermore, estimating the burden attributable to MDD as a risk factor for other health outcomes allows for a more accurate estimate of burden and reinforces the importance of implementing cost-effectiveness interventions to reduce its ubiquitous burden. Ongoing improvements to the GBD methodology and access to more epidemiological data will enhance the precision of our burden estimates and add to our understanding of the global burden of depressive disorders.

## Supporting Information

Figure S1 **llustration of the GBD 2010 publications hierarchy.**
(TIF)

Figure S2 **YLD rates (per 100,000) by country for depressive disorders in 1990.**
(TIF)

Table S1 **Summary of epidemiological data sources included for depressive disorders.**
(DOCX)

Table S2 **Point prevalence (%) by region and country for depressive disorders in 2010.**
(DOCX)

Table S3 **Regional DALY and YLD rankings with 95% uncertainty intervals for depressive disorders in 1990.**
(DOCX)

## Abbreviations

CRA: comparative risk assessment
DALY: disability adjusted life years
DSM: Diagnostic and Statistical Manual of Mental Disorders
GBD: global burden of disease
ICD: International Classification of Diseases
MDD: major depressive disorder
MEPS: US Medical Expenditure Panel Survey
NESARC: US National Epidemiological Survey on Alcohol and Related Conditions 2000–2001 and 2004–2005
NSMHWB: Australian National Survey of Mental Health and Wellbeing of Adults 1997
RR: relative risk
YLD: years lived with disability
YLL: years of life lost
