# Supplementary figures and images for: Burden of Depressive Disorders by Country, Sex, Age, and Year: Findings from the Global Burden of Disease Study 2010
Source: PLoS Med. 2013 Nov 5;10(11):e1001547. doi: 10.1371/journal.pmed.1001547 (PMC3818162; doi:10.1371/journal.pmed.1001547)

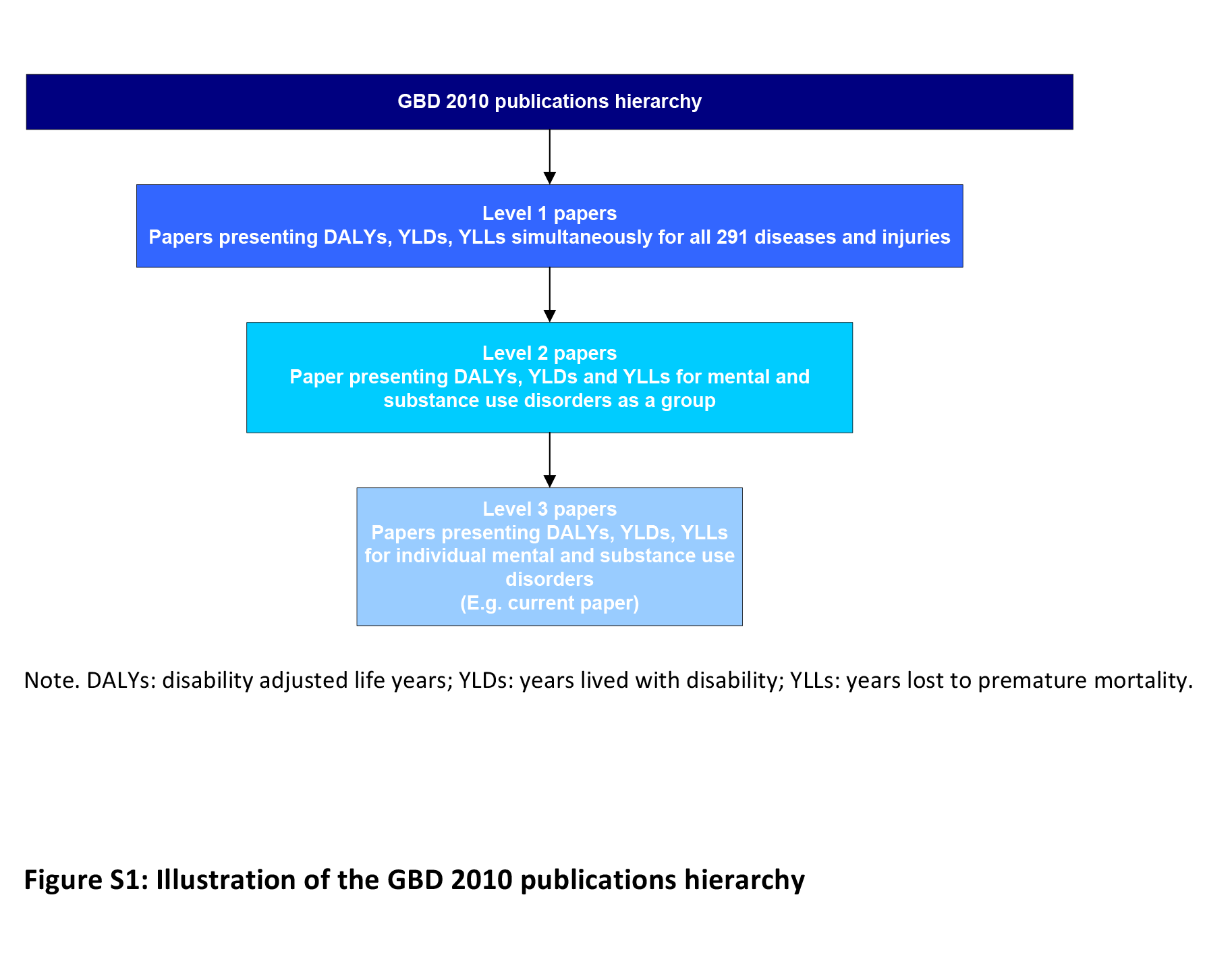

Supplement: Figure S1 — llustration of the GBD 2010 publications hierarchy. (TIF) [file pmed.1001547.s001.tif]

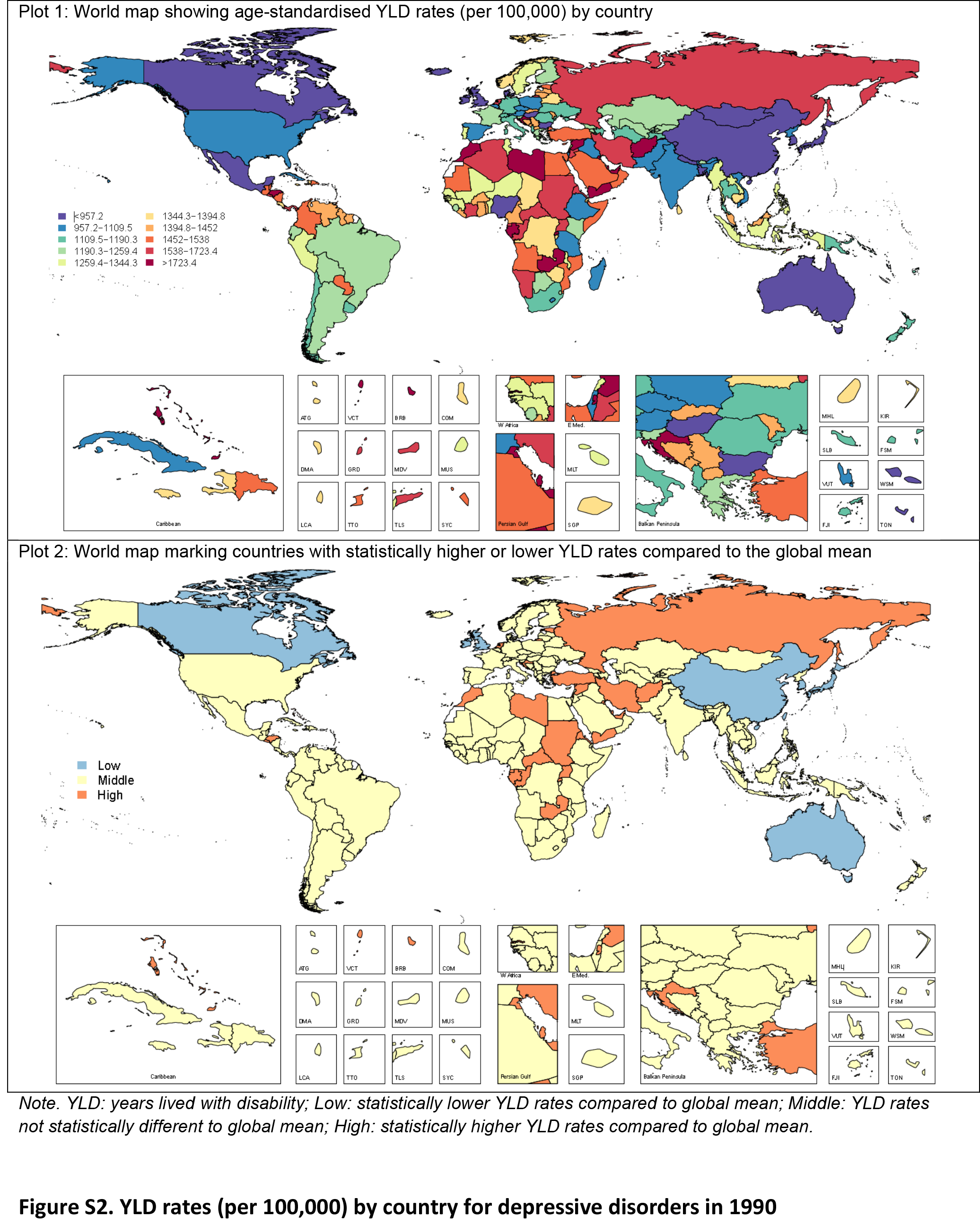

Supplement: Figure S2 — YLD rates (per 100,000) by country for depressive disorders in 1990. (TIF) [file pmed.1001547.s002.tif]
