# Supplementary material for: Burden of Depressive Disorders by Country, Sex, Age, and Year: Findings from the Global Burden of Disease Study 2010
Source: PLoS Med. 2013 Nov 5;10(11):e1001547. doi: 10.1371/journal.pmed.1001547 (PMC3818162; doi:10.1371/journal.pmed.1001547)
Supplement: Table S1 — Summary of epidemiological data sources included for depressive disorders. (DOCX) [file pmed.1001547.s003.docx]

**Table S1: Summary of epidemiological data obtained from the systematic review of the literature and included in the DisMod-MR modelling of major depressive disorder and dysthymia.**

|  | **Major depressive disorder** | | | | | **Dysthymia** | | | | |
| --- | --- | --- | --- | --- | --- | --- | --- | --- | --- | --- |
| **Region** | **Studies** | **Number of estimates** | | | | **Studies** | **Number of estimates** | | | |
|  |  | **Prevalence** | **Incidence** | **Duration** | **Excess-mortality** |  | **Prevalence** | **Incidence** | **Remission** | **Excess-mortality** |
| **Asia Pacific, High Income** | [[1-8](#_ENREF_1)] | 15 | - | - | - | [[1-8](#_ENREF_1)] | 12 | - | - | - |
| **Asia, Central** | - | - | - | - | - | - | - | - | - | - |
| **Asia, East** | [[6](#_ENREF_6),[9-16](#_ENREF_9)] | 59 | - | - | - | [[9-12](#_ENREF_9)] | 19 | - | - | - |
| **Asia, South** | [[6](#_ENREF_6),[17](#_ENREF_17),[18](#_ENREF_18) ,[19](#_ENREF_19) ] | 6 | - | - | - | - | - | - | - | - |
| **Asia, Southeast** | [[20](#_ENREF_20)] | 16 | - | - | - | - | - | - | - | - |
| **Australasia** | [[21-30](#_ENREF_21)] | 41 | - | - | 1 | [[21-30](#_ENREF_21)] | 13 | - | - | - |
| **Caribbean** | [[31](#_ENREF_31),[32](#_ENREF_32)] | 6 | - | - | - | [[31](#_ENREF_31),[32](#_ENREF_32)] | 1 | - | - | - |
| **Europe, Central** | [[33-35](#_ENREF_33)] | 23 | - | - | - | - | - | - | - | - |
| **Europe, Eastern** | [[36-38](#_ENREF_36)] | 13 | - | - | - | [[36-38](#_ENREF_36)] | 6 | - | - | - |
| **Europe, Western** | [[6](#_ENREF_6),[7](#_ENREF_7),[35](#_ENREF_35),[39-74](#_ENREF_39)] | 170 | - | 1 | 6 | [[50](#_ENREF_50),[57](#_ENREF_57),[61-67](#_ENREF_61),[75](#_ENREF_75),[76](#_ENREF_76)] | 35 | - | 2 | 0 |
| **Latin America, Andean** | - | - | - | - | - | - | - | - | - | - |
| **Latin America, Central** | [[35](#_ENREF_35),[77-80](#_ENREF_77)] | 8 | - | - | - | [[79](#_ENREF_79),[80](#_ENREF_80)] | 2 | - | - | - |
| **Latin America, Southern** | [[6](#_ENREF_6),[35](#_ENREF_35),[81](#_ENREF_81)] | 4 | - | - | - | - | - | - | - | - |
| **Latin America, Tropical** | [[6](#_ENREF_6),[82-84](#_ENREF_82)] | 11 | - | - | - | [[6](#_ENREF_6),[82-84](#_ENREF_82)] | 8 | - | - | - |
| **North Africa/Middle East** | [[6](#_ENREF_6),[35](#_ENREF_35),[85-92](#_ENREF_85)] | 22 | - | - | - | {[[6](#_ENREF_6),[35](#_ENREF_35),[85-92](#_ENREF_85)] | 3 | - | - | - |
| **North America, High Income** | [[6](#_ENREF_6),[35](#_ENREF_35),[93-116](#_ENREF_93)] | 122 | 18 | 4 | 6 | [[94](#_ENREF_94),[99](#_ENREF_99),[100](#_ENREF_100),[102](#_ENREF_102),[104](#_ENREF_104),[111](#_ENREF_111),[117](#_ENREF_117)] | 38 | 3 | 1 | - |
| **Oceania** | - | - | - | - | - | - | - | - | - | - |
| **Sub-Saharan Africa, Central** | - | - | - | - | - | - | - | - | - | - |
| **Sub-Saharan Africa, East** | [[118-123](#_ENREF_118)] | 9 | 1 | - | 1 | [[118-123](#_ENREF_118)] | 2 | - | - | - |
| **Sub-Saharan Africa, Southern** | [[124](#_ENREF_124),[125](#_ENREF_125)] | 3 | - | - | - | [[125](#_ENREF_125)] | 1 | - | - | - |
| **Sub-Saharan Africa, West** | [[6](#_ENREF_6),[126-130](#_ENREF_126)] | 16 | - | - | - | [[126](#_ENREF_126)] | 1 | - | - | - |

*Note. Some studies reported more than one estimate.*

**References**

1. Cho MJ, Kim JK, Jeon HJ, Suh T, Chung IW, et al. (2007) Lifetime and 12-month prevalence of DSM-IV psychiatric disorders among Korean adults. Journal of Nervous and Mental Disease 195: 203-210.

2. Kawakami N, Shimizu H, Haratani T, Iwata N, Kitamura T (2004) Lifetime and 6-month prevalence of DSM-III-R psychiatric disorders in an urban community in Japan. Psychiatry Research 121: 293-301.

3. Kawakami N, Takeshima T, Ono Y, Uda H, Hata Y, et al. (2005) Twelve-month prevalence, severity, and treatment of common mental disorders in communities in Japan: Preliminary finding from the World Mental Health Japan Survey 2002-2003. Psychiatry and Clinical Neurosciences 59: 441-452.

4. Nakao M, Yano E (2006) Somatic symptoms for predicting depression: One-year follow-up study in annual health examinations. Psychiatry & Clinical Neurosciences 60: 219–225.

5. Ihara K, Muraoka Y, Oiji A, Nadaoka T (1998) Prevalence of mood disorders according to DSM-III-R criteria in the community elderly residents in Japan. Environmental Health and Preventive Medicine 3: 44–49.

6. Simon GE, Goldberg D, Von Korff M, Ustun T (2002) Understanding cross-national differences in depression prevalence. Psychological Medicine 32: 585–594.

7. Weissman MM, Bland RC, Canino GJ, Faravelli C, Greenwald S, et al. (1996) Cross-national epidemiology of major depression and bipolar disorder. JAMA: The Journal of the American Medical Association 276: 293–299.

8. Fones CS, Kua EH, Ng TP, Ko SM (1998) Studying the mental health of a nation: a preliminary report on a population survey in Singapore. Singapore Medical Journal 39: 251–255.

9. Phillips MR, Zhang J, Shi Q, Song Z, Ding Z, et al. (2009) Prevalence, treatment, and associated disability of mental disorders in four provinces in China during 2001–05: an epidemiological survey. The Lancet 373: 2041–2053.

10. Keqing L, Ze C, Lijun C, Qinpu J, Guang S, et al. (2008) Epidemiological survey of mental disorders in the people aged 18 and older in Hebei province. Asian Journal of Psychiatry 1: 51–55.

11. Shen Y, Zhang M, Huang Y, He Y, Liu Z, et al. (2006) Twelve-month prevalence, severity and unmet need for treatment of mental disorders in metropolitan China. Psychological Medicine 36: 257-267.

12. Yang HJ, Soong WT, Kuo PH, Chang HL, Chen WJ (2004) Using the CES-D in a two-phase survey for depressive disorders among nonreferred adolescents in Taipei: A stratum-specific likelihood ratio analysis. Journal of Affective Disorders 82: 419–430.

13. Chen R, Hu Z, Qin X, Xu X, Copeland JRM (2004) A community-based study of depression in older people in Hefei, China – The GMS-AGECAT prevalence, case validation and socio-economic correlates. International Journal of Geriatric Psychiatry 19: 407–413.

14. Hwu H-G, Chang IH, Yeh E-K, Chang C-J, Yeh L-L (1996) Major depressive disorder in Taiwan defined by the Chinese Diagnostic Interview Schedule. Journal of Nervous and Mental Disease 184: 497–502.

15. Lu J, Ruan Y, Huang Y, Yao J, Dang W, et al. (2008) Major depression in Kunming: Prevalence, correlates and co-morbidity in a south-western city of China. Journal of Affective Disorders 111: 221–226.

16. Chong M-Y, Chen C-C, Tsang H-Y, Yeh T-L, Chen C-S, et al. (2001) Community study of depression in old age in Taiwan: Prevalence, life events and socio-demographic correlates. British Journal of Psychiatry 178: 29–35.

17. Nisar N, Billoo N, Gadit AA (2004) Prevalence of depression and the associated risks factors among adult women in a fishing community. Journal of the Pakistan Medical Association 54: 519–525.

18. Srinath S, Girimaji S, S S, Subbakrishna D, Bhola P, et al. (2005) Epidemiological study of child and adolescent psychiatric disorders in urban and rural areas of Bangalore India. Indian Journal of Medical Research 122: 67–79.

19. Subedi S, Tausig M, Subedi J, Broughton CL, Williams-Blangero S (2004) Mental Illness and Disability among Elders in Developing Countries: The Case of Nepal. Journal of Aging and Health 16: 71–87.

20. Nguyen TTN, Long TK, Bui NL, Vos T, Ngo DA, et al. (2011) Viet Nam Burden of Disease and Injury Study. Hanoi.

21. Feehan M, McGee R, Raja SN, Williams SM (1994) DSM-III-R disorders in New Zealand 18-year-olds. Australian and New Zealand Journal of Psychiatry 28: 87–99.

22. Kashani JH, McGee RO, Clarkson SE, Anderson JC, Walton LA, et al. (1983) Depression in a sample of 9-year-old children: Prevalence and associated characteristics. Archives of General Psychiatry 40: 1217–1223.

23. McGee R, Feehan M, Williams S, Partridge F, Silva PA, et al. (1990) DSM-III disorders in a large sample of adolescents. Journal of the American Academy of Child and Adolscent Psychiatry 29: 611-619.

24. Sawyer MG, Miller-Lewis LR, Clark JJ (2007) The mental health of 13-17 year-olds in Australia: Findings from the National Survey of Mental Health and Well-Being. Journal of Youth Adolescence 36: 185–194.

25. Wilhelm K, Mitchell P, Slade T, Brownhill S, Andrews G (2003) Prevalence and correlates of DSM-IV major depression in an Australian national survey. Journal of Affective Disorders 75: 155–162.

26. Hawthorne G, Goldney R, Taylor AW (2008) Depression prevalence: Is it really increasing? . The Australian and New Zealand Journal of Psychiatry 42: 606–616.

27. Wells JE, Oakley Browne MA, Scott KM, McGee MA, Baxter J, et al. (2006) Prevalence, interference with life and severity of 12 month DSM-IV disorders in Te Rau Hinengaro: The New Zealand Mental Health Survey. Australian and New Zealand Journal of Psychiatry 40: 845–854.

28. Australian Bureau of Statistics (2008) National Survey of Mental Health and Wellbeing: Summary of results; ABS, editor. Canberra: Australian Bureau of Statistics.

29. Fergusson DM, Horwood LJ, Lynskey MT (1993) Prevalence and comorbidity of DSM-III-R diagnoses in a birth cohort of 15 year olds. Journal of the American Academy of Child and Adolescent Psychiatry 32: 1127–1134.

30. Jorm A, Henderson A, Kay D, Jacomb P (1991) Mortality in relation to dementia, depression and social integration in an elderly community sample. International Journal of Geriatric Psychiatry 6: 5-11.

31. Canino GJ, Bird HR, Shrout PE, Rubio-Stipec M, Bravo M, et al. (1987) The prevalence of specific psychiatric disorders in Puerto Rico. Archives Of General Psychiatry 44: 727–735.

32. Canino G, Shrout PE, Rubio-Stipec M, Bird HR, Bravo M, et al. (2004) The DSM-IV Rates of child and adolescent disorders in Puerto Rico: Prevalence, correlates, service use, and the effects of impairment. Archives of General Psychiatry 61: 85–93.

33. Basoglu M, Livanou M, Crnobaric C, Franciskovic T, Suljic E, et al. (2005) Psychiatric and cognitive effects of war in former Yugoslavia: Association of lack of redress for trauma and posttraumatic stress reactions. JAMA: The Journal of the American Medical Association 294: 580–590.

34. Szadoczky E, Papp Z, Vitrai J, Rihmer Z, Furedi J (1998) The prevalence of major depressive and bipolar disorders in Hungary. Results from a national epidemiologic survey. Journal of Affective Disorders 50: 153–162.

35. Andrade L, Caraveo-Anduaga JJ, Berglund P, Bijl RV, De Graaf R, et al. (2003) The epidemiology of major depressive episodes: Results from the International Consortium of Psychiatric Epidemiology (ICPE) Surveys. International Journal of Methods in Psychiatric Research 12: 3–21.

36. Pakriev S, Vasar V, Aluoja A, Saarma M, Shlik J (1998) Prevalence of mood disorders in the rural population of Udmurtia. Acta Psychiatrica Scandinavica 97: 169–174.

37. Aluoja A, Leinsalu M, Shlik J, Vasar V, Luuk K (2004) Symptoms of depression in the Estonian population: prevalence, sociodemographic correlates and social adjustment. Journal of Affective Disorders 78: 27–35.

38. Bromet EJ, Gluzman SF, Paniotto VI, Webb CPM, Tintle NL, et al. (2005) Epidemiology of psychiatric and alcohol disorders in Ukraine: Findings from the Ukraine World Mental Health Survey. Social Psychiatry and Psychiatric Epidemiology 40: 681-690.

39. Beekman ATF, Deeg DJJ, Van Tilburg T, Smith JH, Hooijer C, et al. (1995) Major and minor depression in later life: A study of prevalence and risk factors. Journal of Affective Disorders 36: 65–75.

40. Stefansson GE, Bjornsson JK, Gudmundsdottir A (1994) Period prevalence rates of specific mental disorders in an Icelandic cohort. Social Psychiatry and Psychiatric Epidemiology 29: 119–125.

41. Kirby M, Bruce I, Radic A, Coakley D, Lawlor BA (1997) Mental disorders among the community-dwelling elderly in Dublin. British Journal of Psychiatry 171: 369–372.

42. Godin O, Dufouil C, Ritchie K, Dartigues JF, Tzourio C, et al. (2007) Depressive symptoms, major depressive episode and cognition in the elderly: The three-city study. Neuroepidemiology 28: 101–108.

43. Green H, McGinnty A, Meltzer H, Ford T, Goodman R (2005) Mental Health of Children and Young People in Great Britain, 2004. UK National Statistics: 1-156.

44. Pahkala K, Kesti E, Kongas-Saviaro P, Laippala P, Kivela SL (1995) Prevalence of depression in an aged population in Finland. Social Psychiatry & Psychiatric Epidemiology 30: 99–106.

45. Ritchie K, Artero S, Beluche I, Ancelin ML, Mann A, et al. (2004) Prevalence of DSM-IV psychiatric disorder in the French elderly population. British Journal of Psychiatry 184: 147–152.

46. Saunders PA, Copeland JRM, Dewey ME, Gilmore C, Larkin BA, et al. (1993) The prevalence of dementia, depression and neurosis in later life: The Liverpool MRC-ALPHA study. International Journal of Epidemiology 22: 838–847.

47. Jenkins R, Lewis G, Bebbinton P, Brugha T, Farrell M, et al. (1997) The National Psychiatric Morbidity Surveys of Great Britain – Initial findings from the Household Survey. . Psychological Medicine 27: 775–789.

48. Singleton N, Bumpstead R, O'Brien M, Lee A, Meltzer H (2001) Psychiatric morbidity among adults living in private households. London: The Stationery Office.

49. Barry MM, Van Lente E, Molcho M, Morgan K, McGee H, et al. (2009) SLAN 2007: Survey of lifestyle, attitudes and nutrition in Ireland. Mental Health and Social Well-being Report. Dublin: Department of Health and Children.

50. Kringlen E, Torgensen S, Cramer V (2001) A Norwegian psychiatric epidemiological study. American Journal of Psychiatry 158: 1091-1098.

51. Bracke P (1998) Sex differences in the course of depression: evidence from a longitudinal study of a representative sample of the Belgian population. Social Psychiatry & Psychiatric Epidemiology 33: 420–429.

52. Levav I, Kohn R, Dohrenwend BP, Shrout PE, Skodol AE, et al. (1993) An epidemiological study of mental disorders in a 10-year cohort of young adults in Israel. Psychological Medicine 23: 691–707.

53. Frojd S, Marttunen M, Pelkonen M, von der Pahlen B, Kaltiala-Heino R (2007) Adult and peer involvement in help-seeking for depression in adolescent population. A two-year follow-up in Finland. Social Psychiatry and Psychiatric Epidemiology 42: 945–952.

54. Jylha P, Isometsa E (2006) The relationship of neuroticism and extraversion to symptoms of anxiety and depression in the general population. Depression & Anxiety 23: 281–289.

55. Meltzer H, R. G, Goodman R, Ford T (2000) The mental health of children and adolescents in Great Britain. London: The Stationery Office

56. Copeland JRM, Beekman ATF, Dewey ME, Hooijer C, Jordan A, et al. (1999) Depression in Europe. Geographical distribution among older people. British Journal of Psychiatry 174: 312–321.

57. Carta MG, Kovess V, Hardoy MC, Morosini P, Murgia S, et al. (2002) Psychiatric disorders in Sardinian immigrants to Paris: A comparison with Parisians and Sardinians resident in Sardinia. Social Psychiatry and Psychiatric Epidemiology 37: 112–117.

58. Lepine J, Gastpar M, Mendlewicz J, Tylee A (1997) Depression in the community: The first pan-European study. DEPRES (Depression Research in European Society). International Clinical Psychopharmacology 12: 19–29.

59. Angst J, Merikangas K, Scheidegger P, Wicki W (1990) Recurrent brief depression: a new subtype of affective disorder. Journal of Affective Disorders 19: 87–98.

60. Ponizovsky AM, Grinshpoon A (2009) Mood and anxiety disorders and the use of services and psychotropic medication in an immigrant population: Findings from the Israel National Health Survey. Canadian Journal of Psychiatry 54: 409–419.

61. Verhulst FC, Van der Ende J, Ferdinand RF, Kasius MC (1997) The prevalence of DSM-III-R diagnoses in a national sample of dutch adolescents. Archives of General Psychiatry 54: 329–336.

62. Oldehinkel AJ, Wittchen HU, Schuster P (1999) Prevalence, 20-month incidence and outcome of unipolar depressive disorders in a community sample of adolescents. Psychological Medicine 29: 655–668.

63. Pirkola SP, Isometsa E, Suvisaari J, Aro H, Joukamaa M, et al. (2005) DSM-IV mood-, anxiety- and alcohol use disorders and their comorbidity in the Finnish general population. Results from the Health 2000 Study. Social Psychiatry and Psychiatric Epidemiology 40: 1-10.

64. Faravelli C, Degl'Innocenti BG, Aiazzi L, Incerpi G, Pallanti S (1990) Epidemiology of mood disorders: A community survey in Florence. Journal of Affective Disorders 20: 135–141.

65. Almqvist F, Puura K, Kumpulainen K, Tuompo-Johansson E, Henttonen I, et al. (1999) Psychiatric disorders in 8–9-year-old children based on a diagnostic interview with the parents. European Child and Adolescent Psychiatry 8: 17–28.

66. Aalto-Setala T, Marttunen M, Tuulio-Henriksson A, Poikolainen K, Lonnqvist J (2001) One-month prevalence of depression and other DSM-IV disorders among young adults. Psychological Medicine 31: 791-801.

67. Ayuso-Mateos JL, Vazques-Barquero JL, Dowrick C, Lehtinen V, Dalgard OS, et al. (2001) Depressive disorders in Europe: Prevalence figures from the ODIN study. British Journal of Psychiatry 179: 308–316.

68. Bergdahl E, Gustavsson JM, Kallin K, von Heideken Wagert P, Lundman B, et al. (2005) Depression among the oldest old: The Umea 85+ study. International Psychogeriatrics 17: 557–575.

69. Pulska T, Pahkala K, Laippalla P, Kivelä SL (1998) Major depression as a predictor of premature deaths in lderly people in Finland: a community study. Acta Psychiatrica Scandinavica 97: 408-411.

70. Adamson JA, Price GM, Breeze E, Bulpitt CJ, Fletcher AE (2005) Are older people dying of depression? Findings from the Medical Research Council trial of the assessment and management of older people in the community. Journal of the American Geriatrics Society 53: 1128-1132.

71. Penninx BWJH, Geerlings SW, Deeg DJH, van Eijk J, van Tilburg W, et al. (1999) Minor and major depression and the risk of death in older persons. Archives of General Psychiatry 56: 889.

72. Vinkers DJ, Stek ML, Gussekloo J, van der Mast RC, Westendorp RGJ (2004) Does depression in old age increase only cardiovascular mortality? The Leiden 85‐plus Study. International Journal of Geriatric Psychiatry 19: 852-857.

73. Donnelly M (1995) Depression among adolescents in Northern Ireland. . Adolescence 30: 339–351.

74. Spijker J, de Graaf R, Bijl RV, Beekman AT, Ormel J, et al. (2002) Duration of major depressive episodes in the general population: results from The Netherlands Mental Health Survey and Incidence Study (NEMESIS). Br J Psychiatry 181: 208-213.

75. Angst J, Wicki W (1991) The Zurich study. XI. Is dysthymia a separate form of depression? Results of the Zurich Cohort Study. European Archives of Psychiatry and Clinical Neuroscience 240: 349–354.

76. Kivela SL, Kongas-Savlaro P, Pahkala K, Kesti E, Laippala P (1993) Five-year prognosis for dysthymic disorder in old age. International Journal of Geriatric Psychiatry 8: 939–947.

77. Slone LB, Norris FH, Murphy AD, Baker CK, Perilla JL, et al. (2006) Epidemiology of major depression in four cities in Mexico. Depression and Anxiety 23: 158–167.

78. Kohn R, Levav I, Garcia ID, Machuca ME, Tamashiro R (2005) Prevalence, risk factors and aging vulnerability for psychopathology following a natural disaster in a developing country. International Journal of Geriatric Psychiatry 20: 835–841.

79. Medina-Mora Icaza ME, Borges-Guimaraes G, Lara C, Ramos-Lira L, Zambrano J, et al. (2005) Prevalence of violent events and post-traumatic stress disorder in the Mexican population. Salud Publica de Mexico 47: 8-22.

80. Benjet C, Borges G, Medina-Mora ME, Zambrano J, Aguilar-Gaxiola S (2009) Youth mental health in a populous city of the developing worlds: results from the Mexican Adolescent Mental health Survey. . The Journal of Child Psychology and Psychiatry 50: 386-395.

81. Araya R, Rojas G, Fritsch R, Acuna J, Lewis G (2001) Common mental disorders in Santiago, Chile: Prevalence and socio-demographic correlates. British Journal of Psychiatry 178: 228–233.

82. Andrade L, Walters E, Gentil V, Laurenti R (2002) Prevalence of ICD-10 mental disorders in a catchment area in the city of Sao Paulo, Brazil. Social Psychiatry and Psychiatric Epidemiology 3: 316–325.

83. Costa E, Barreto SM, Uchoa E, Firmo JOA, Lima-Costa MF, et al. (2007) Prevalence of International Classification of Diseases, 10th Revision common mental disorders in the elderly in a Brazilian community: The Bambui health ageing study. American Journal of Geriatric Psychiatry 15: 17–27.

84. Fleitlich-Bilyk B, Goodman R (2004) Prevalence of child and adolescent psychiatric disorders in southeast Brazil. Journal of the American Academy of Child & Adolescent Psychiatry 43: 727–734.

85. Lopes Cardozo B, Bilukha OO, Gotway CA, Wolfe MI, Gerber ML, et al. (2005) Mental health of women in postwar Afghanistan. Journal of Women's Health 14: 285-293.

86. Scholte WF, Olff M, Ventevogel P, De Vries GJ, Jansveld E, et al. (2004) Mental health symptoms following war and repression in Eastern Afghanistan. Journal of the American Medical Association 292: 585–593.

87. Afifi M, Al Riyami A, Morsi M, Al Kharusil H, Afifi M, et al. (2006) Depressive symptoms among high school adolescents in Oman. Eastern Mediterranean Health Journal 12 Suppl 2: S126–S137.

88. Bostanci M, Ozdel O, Oguzhanoglu NK, Ozdel L, Ergin A, et al. (2005) Depressive symptomatology among university students in Denizli, Turkey: prevalence and sociodemographic correlates. Croatian Medical Journal 46: 96–100.

89. Ghanem M, Gadallah M, Meky FA, Mourad S, El-Kholy G (2009) National survey of prevalence of mental disorders in Egypt: Preliminary survey. Eastern Mediterranean Health Journal 15: 65–75.

90. Karam EG, Mneimneh ZN, Karam AN, Fayyad JA, Nasser SC, et al. (2006) Prevalence and treatment of mental disorders in Lebanon: A national epidemiological survey. The Lancet 367: 1000–1006.

91. Alhasnawi A, Sadik S, Rasheed M, Baban A, Al-Alak MM, et al. (2009) The prevalence and correlates of DSM-IV disorders in the Iraq mental health survey (IHMS). World Psychiatry 8: 97–109.

92. Al-Jawadi A, Abdul-Rhman S (2007) Prevalence of childhood and early adolescence mental disorders among children attending primary health care centres in Mosul, Iraq: A cross-sectional study. BMC Public Health 7: 274–281.

93. Bland RC, Newman SC, Orn H (1997) Age and remission of psychiatric disorders. Canadian Journal of Psychiatry 42: 722–729.

94. Bland R, Newman S, Orn H (1988) Period prevalence of psychiatric disorders in Edmonton. Acta Psychiatrica Scandinavica 77: 33–42.

95. Blazer DG, Kessler RC, McGonagle KA, Swartz MS (1994) The prevalence and distribution of major depression in a national community sample: the National Comorbidity Survey. Am J Psychiatry 151: 979-986.

96. Cohen P, Cohen J, Kasen S, Velez CN, Hartmark C, et al. (1993) An epidemiological study of disorders in late childhood and adolescence – I. Age- and gender-specific prevalence. Journal of Child Psychology and Psychiatry and Allied Disciplines 34: 851–867.

97. Costello EJ, Angold A, Burns BJ, Erkanli A, Stangl DK, et al. (1996) The Great Smoky Mountains Study of youth: Functional impairment and serious emotional disturbance. Archives of General Psychiatry 53: 1137–1143.

98. Fleming JE, Offord DR, Boyle MH (1989) Prevalence of childhood and adolescent depression in the community: Ontario Child Health Study. British Journal of Psychiatry 155: 647–654.

99. Garrison CZ, Addy CL, Jackson KL, McKeown RE, Waller JL (1992) Major depressive disorder and dysthymia in young adolescents. American Journal of Epidemiology 135: 792–802.

100. Gum AM, King-Kallimanis B, Kohn R (2009) Prevalence of mood, anxiety, and substance-abuse disorders for older Americans in the National Comorbidity Survey-replication. The American Journal of Geriatric Psychiatry 17: 769–781.

101. Kessler RC, McGonagle KA, Swartz M, Blazer DG, Nelson CB (1993) Sex and depression in the National Comorbidity Survey: I. Lifetime prevalence, chronicity and recurrence. Journal of Affective Disorders 29: 85–96.

102. Kessler RC, McGonable KA, Zao S, Nelson CB, Hughes M, et al. (1994) Lifetime and 12-month prevalence of DSM-III-R psychiatric disorders in the United States; results from the National Comorbidity Survey. Archives of General Psychiatry 51: 8-19.

103. Kessler RC, Walters EE (1998) Epidemiology of DSM-III-R major depression and minor depression among adolescents and young adults in the National Comorbidity Survey. Depression and Anxiety 7: 3–14.

104. Lewinsohn PM, Hops H, Roberts RE, Seeley JR, Andrews JA (1993) Adolescent psychopathology: I. Prevalence and incidence of depression and other DSM-III-R disorders in high school students. Journal of Abnormal Psychology 102: 133–144.

105. Mojtabai R, Olfson M (2004) Major depression in community-dwelling middle-aged and older adults: prevalence and 2- and 4-year follow-up symptoms. Psychological Medicine 34: 623–634.

106. Newman SC, Sheldon CT, Bland RC (1998) Prevalence of depression in an elderly community sample: a comparison of GMS-AGECAT and DSM-IV diagnostic criteria. Psychological Medicine 28: 1339–1345.

107. Newman SC, Bland RC, Orn HT (1998) The prevalence of mental disorders in the elderly in Edmonton: a community survey using GMS-AGECAT. Geriatric Mental State-Automated Geriatric Examination for Computer Assisted Taxonomy. Canadian Journal of Psychiatry 43: 910–914.

108. Offord DR, Boyle MH, Campbell D, Goering P, Lin E, et al. (1996) One-year prevalence of psychiatric disorder in Ontarians 15 to 64 years of age. . Canadian Journal of Psychiatry 41: 559–563.

109. Patten SB (2001) The duration of major depressive episodes in the Canadian general population. Chronic Diseases in Canada 22: 6–11.

110. Patten SB, Stuart HL, Russell ML, Maxwell CJ, Arboleda-Florez J (2003) Epidemiology of major depression in a predominantly rural health region. Social Psychiatry and Psychiatric Epidemiology 38: 360–365.

111. Regier DA, Boyd JH, Burke JD, Jr., Rae DS, Myers JK, et al. (1988) One-month prevalence of mental disorders in the United States. Based on five Epidemiologic Catchment Area sites. Archives Of General Psychiatry 45: 977–986.

112. Eaton WW, Kramer M, Anthony JC, Dryman A, Shapiro S, et al. (1989) The incidence of specific DIS/DSM-III mental disorders: data from the NIMH Epidemiologic Catchment Area Program. Acta Psychiatrica Scandinavica 79: 163–178.

113. Gallo JJ, Bogner HR, Morales KH, Post EP, Ten Have T, et al. (2005) Depression, cardiovascular disease, diabetes, and 2-year mortality among older primary care patients. The American journal of geriatric psychiatry: official journal of the American Association for Geriatric Psychiatry 13: 748.

114. Zheng D, Macera CA, Croft JB, Giles WH, Davis D, et al. (1997) Major depression and all-cause mortality among white adults in the United States. Annals of Epidemiology 7: 213-218.

115. Bruce ML, Leaf PJ, Rozal GP, Florio L, Hoff RA (1994) Psychiatric status and 9-year mortality data in the New Haven Epidemiologic Catchment Area Study. The American Journal of Psychiatry 151: 716–721.

116. Murphy JM, Monson RR, Olivier DC, Sobol AM, Leighton AH (1987) Affective disorders and mortality: a general population study. Archives of General Psychiatry 44: 473-480.

117. Klein DN, Shankman SA, Rose S (2006) Ten-year prospective follow-up study of the naturalistic course of dysthymic disorder and double depression. American Journal of Psychiatry 163: 872–880.

118. Bolton P, Neugebauer R, Ndogoni L (2002) Prevalence of depression in rural Rwanda based on symptom and functional criteria. Journal of Nervous and Mental Disease 190: 631–637.

119. Ovuga E, Boardman J, Wasserman D (2005) The prevalence of depression in two districts of Uganda. Social Psychiatry and Psychiatric Epidemiology 40: 439–445.

120. Kebede D, Alem A (1999) Major mental disorders in Addis Ababa, Ethiopia. II. Affective disorders. Acta Psychiatrica Scandinavica 100: 18–23.

121. Bolton P, Wilk CM, Ndogoni L (2004) Assessment of depression prevalence in rural Uganda using symptom and function criteria. Social Psychiatry & Psychiatric Epidemiology 39: 442–447.

122. Shaaban KMA, Baashar TA (2003) A community study of depression in adolescent girls: Prevalence and its relation to age. Medical Principles and Practice 12: 256–259.

123. Mogga S, Prince M, Alem A, Kebede D, Stewart R, et al. (2006) Outcome of major depression in Ethiopia: population-based study. British Journal of Psychiatry 189: 241–246.

124. Hollifield M, Katon W, Spain D, Pule L (1990) Anxiety and depression in a village in Lesotho, Africa: A comparison with the United States. British Journal of Psychiatry 156: 343–350.

125. Bhagwanjee A, Parekh A, Paruk Z, Petersen I, Subedar H (1998) Prevalence of minor psychiatric disorders in an adult African rural community in South Africa. Psychological Medicine 28: 1137–1147.

126. Gureje O, Lasebikan VO, Kola L, Makanjuola VA (2006) Lifetime and 12-month prevalence of mental disorders in the Nigerian Survey of Mental Health and Well-Being. British Journal of Psychiatry 188: 465–471.

127. Adewuya AO, Ola BA, Aloba OO, Mapayi BM, Oginni OO (2006) Depression amongst Nigerian university students. Prevalence and sociodemographic correlates. Social Psychiatry & Psychiatric Epidemiology 41: 674–678.

128. Adewuya AO, Ologun YA (2006) Factors associated with depressive symptoms in Nigerian adolescents. Journal of Adolescent Health 39: 105–110.

129. Amoran O, Lawoyin T, Lasebikan V (2007) Prevalence of depression among adults in Oyo State, Nigeria: A comparative study of rural and urban communities. Australian Journal of Rural Health 15: 211–215.

130. Coleman R, Morison L, Paine K, Powell RA, Walraven G (2006) Women's reproductive health and depression: A community survey in the Gambia, West Africa. Social Psychiatry and Psychiatric Epidemiology 41: 720–727.
