# Supplementary material for: Burden of Depressive Disorders by Country, Sex, Age, and Year: Findings from the Global Burden of Disease Study 2010
Source: PLoS Med. 2013 Nov 5;10(11):e1001547. doi: 10.1371/journal.pmed.1001547 (PMC3818162; doi:10.1371/journal.pmed.1001547)
Supplement: Table S2 — Point prevalence (%) by region and country for depressive disorders in 2010. (DOCX) [file pmed.1001547.s004.docx]

**Table S2:** Age standardised point prevalence (%) by region and country for major depressive disorder(MDD) and dysthymia in 2010.

|  | | **MDD** | | | **Dysthymia** | | |
| --- | --- | --- | --- | --- | --- | --- | --- |
| **Region** | **Country** | **Prevalence** | **95% uncertainty interval** | | **Prevalence** | **95% uncertainty interval** | |
| **Global** |  | **4.37%** | **4.09%** | **4.67%** | **1.55%** | **1.50%** | **1.61%** |
| **Asia Pacific, High Income** | | **2.54%** | **2.06%** | **3.07%** | **1.32%** | **1.23%** | **1.42%** |
|  | Brunei | 4.36% | 2.92% | 6.43% | 1.36% | 1.15% | 1.62% |
|  | Japan | 2.46% | 1.88% | 3.08% | 1.31% | 1.20% | 1.42% |
|  | Korea, Rep. | 2.48% | 1.83% | 3.28% | 1.36% | 1.25% | 1.47% |
|  | Singapore | 5.74% | 3.97% | 8.31% | 1.41% | 1.31% | 1.53% |
| **Asia, Central** | | **4.93%** | **4.17%** | **5.85%** | **1.62%** | **1.48%** | **1.78%** |
|  | Armenia | 4.99% | 3.40% | 7.15% | 1.65% | 1.37% | 1.95% |
|  | Azerbaijan | 4.19% | 2.87% | 5.88% | 1.63% | 1.38% | 1.92% |
|  | Georgia | 4.88% | 3.38% | 6.94% | 1.62% | 1.36% | 1.93% |
|  | Kazakhstan | 4.19% | 2.89% | 5.92% | 1.63% | 1.37% | 1.92% |
|  | Kyrgyz Republic | 4.79% | 3.25% | 6.87% | 1.62% | 1.38% | 1.92% |
|  | Mongolia | 4.78% | 3.14% | 6.95% | 1.63% | 1.38% | 1.91% |
|  | Tajikistan | 4.98% | 3.43% | 6.96% | 1.62% | 1.37% | 1.90% |
|  | Turkmenistan | 6.19% | 4.10% | 8.85% | 1.62% | 1.37% | 1.91% |
|  | Uzbekistan | 5.48% | 3.83% | 7.89% | 1.62% | 1.36% | 1.92% |
| **Asia, East** | | **3.00%** | **2.44%** | **3.65%** | **1.58%** | **1.50%** | **1.67%** |
|  | China | 3.02% | 2.43% | 3.68% | 1.58% | 1.50% | 1.67% |
|  | Dem. Peoples Rep. of Korea | 2.61% | 1.75% | 3.77% | 1.61% | 1.37% | 1.89% |
|  | Seychelles | 5.21% | 3.47% | 7.51% | 1.64% | 1.37% | 1.90% |
|  | Taiwan | 2.50% | 1.91% | 3.21% | 1.60% | 1.35% | 1.88% |
| **Asia, South** | | **4.06%** | **3.21%** | **5.05%** | **1.56%** | **1.36%** | **1.78%** |
|  | Bangladesh | 3.35% | 2.30% | 4.67% | 1.56% | 1.31% | 1.84% |
|  | Bhutan | 3.83% | 2.56% | 5.53% | 1.54% | 1.30% | 1.79% |
|  | India | 4.06% | 3.05% | 5.29% | 1.56% | 1.32% | 1.84% |
|  | Nepal | 3.04% | 2.21% | 4.11% | 1.57% | 1.34% | 1.86% |
|  | Pakistan | 4.83% | 3.61% | 6.50% | 1.56% | 1.32% | 1.82% |
| **Asia, Southeast** | | **4.90%** | **4.10%** | **5.87%** | **1.56%** | **1.36%** | **1.78%** |
|  | Indonesia | 4.90% | 3.36% | 7.00% | 1.63% | 1.37% | 1.91% |
|  | Cambodia | 4.88% | 3.39% | 6.90% | 1.65% | 1.39% | 1.92% |
|  | Lao PDR | 5.81% | 3.99% | 8.32% | 1.64% | 1.37% | 1.93% |
|  | Sri Lanka | 4.73% | 3.19% | 6.66% | 1.63% | 1.38% | 1.93% |
|  | Maldives | 5.08% | 3.39% | 7.37% | 1.62% | 1.38% | 1.88% |
|  | Myanmar | 5.16% | 3.57% | 7.21% | 1.63% | 1.36% | 1.91% |
|  | Mauritius | 5.13% | 3.42% | 7.43% | 1.63% | 1.38% | 1.88% |
|  | Malaysia | 5.57% | 3.72% | 7.87% | 1.63% | 1.38% | 1.93% |
|  | Philippines | 5.22% | 3.54% | 7.46% | 1.63% | 1.40% | 1.90% |
|  | Thailand | 5.20% | 3.57% | 7.25% | 1.64% | 1.37% | 1.93% |
|  | Timor Leste | 5.96% | 4.00% | 8.71% | 1.62% | 1.36% | 1.91% |
|  | Viet Nam | 3.99% | 2.99% | 5.21% | 1.64% | 1.40% | 1.94% |
| **Australasia** | | **3.32%** | **2.82%** | **3.90%** | **1.51%** | **1.41%** | **1.62%** |
|  | Australia | 3.05% | 2.51% | 3.69% | 1.51% | 1.41% | 1.63% |
|  | New Zealand | 4.65% | 3.58% | 5.84% | 1.48% | 1.38% | 1.59% |
| **Caribbean** | | **5.16%** | **4.29%** | **6.21%** | **1.54%** | **1.41%** | **1.71%** |
|  | Antigua and Barbuda | 7.07% | 4.61% | 10.62% | 1.53% | 1.29% | 1.79% |
|  | Bahamas | 5.42% | 3.61% | 7.76% | 1.55% | 1.33% | 1.83% |
|  | Belize | 5.41% | 3.66% | 7.59% | 1.54% | 1.29% | 1.80% |
|  | Barbados | 5.32% | 3.58% | 7.66% | 1.54% | 1.30% | 1.84% |
|  | Cuba | 5.84% | 3.99% | 8.34% | 1.54% | 1.32% | 1.85% |
|  | Dominica | 5.40% | 3.71% | 7.73% | 1.55% | 1.31% | 1.82% |
|  | Dominican Republic | 4.48% | 3.05% | 6.28% | 1.54% | 1.29% | 1.81% |
|  | Grenada | 5.41% | 3.65% | 7.78% | 1.54% | 1.29% | 1.81% |
|  | Guyana | 6.29% | 4.20% | 9.24% | 1.54% | 1.31% | 1.79% |
|  | Haiti | 4.84% | 3.28% | 6.89% | 1.54% | 1.29% | 1.82% |
|  | Jamaica | 4.79% | 3.34% | 6.93% | 1.55% | 1.31% | 1.82% |

|  | | **MDD** | | | **Dysthymia** | | |
| --- | --- | --- | --- | --- | --- | --- | --- |
| **Region** | **Country** | **Prevalence** | **95% uncertainty interval** | | **Prevalence** | **95% uncertainty interval** | |
| **Caribbean** | | **5.16%** | **4.29%** | **6.21%** | **1.54%** | **1.41%** | **1.71%** |
|  | St Lucia | 4.25% | 2.82% | 6.02% | 1.54% | 1.31% | 1.81% |
|  | Suriname | 5.37% | 3.54% | 7.78% | 1.54% | 1.31% | 1.80% |
|  | Trinidad and Tobago | 5.50% | 3.64% | 7.78% | 1.56% | 1.31% | 1.84% |
|  | Saint Vincent and Grenadines | 5.36% | 3.66% | 7.53% | 1.53% | 1.30% | 1.80% |
| **Europe, Central** | | **4.09%** | **3.51%** | **4.79%** | **1.62%** | **1.48%** | **1.77%** |
|  | Albania | 5.17% | 3.48% | 7.45% | 1.62% | 1.37% | 1.90% |
|  | Bulgaria | 4.58% | 3.09% | 6.54% | 1.62% | 1.34% | 1.92% |
|  | Bosnia & Herzegovina | 3.56% | 2.47% | 5.17% | 1.61% | 1.35% | 1.88% |
|  | Czech Republic | 3.23% | 2.28% | 4.36% | 1.61% | 1.36% | 1.91% |
|  | Croatia | 7.06% | 5.06% | 9.67% | 1.62% | 1.38% | 1.92% |
|  | Hungary | 3.31% | 2.59% | 4.13% | 1.62% | 1.38% | 1.92% |
|  | Poland | 3.91% | 2.70% | 5.56% | 1.62% | 1.36% | 1.90% |
|  | Romania | 4.28% | 2.99% | 5.99% | 1.61% | 1.36% | 1.88% |
|  | Serbia | 4.01% | 2.68% | 5.76% | 1.62% | 1.36% | 1.90% |
|  | Slovak Republic | 3.60% | 2.44% | 5.13% | 1.62% | 1.38% | 1.91% |
|  | Slovenia | 4.52% | 3.02% | 6.56% | 1.62% | 1.39% | 1.92% |
|  | Macedonia | 5.23% | 3.57% | 7.56% | 1.62% | 1.35% | 1.93% |
|  | Montenegro | 6.02% | 3.90% | 9.07% | 1.61% | 1.36% | 1.90% |
| **Europe, Eastern** | | **5.88%** | **4.51%** | **7.63%** | **1.59%** | **1.50%** | **1.70%** |
|  | Belarus | 6.56% | 4.42% | 9.49% | 1.60% | 1.35% | 1.87% |
|  | Estonia | 6.75% | 5.15% | 8.79% | 1.61% | 1.36% | 1.90% |
|  | Lithuania | 4.79% | 3.27% | 6.53% | 1.60% | 1.36% | 1.89% |
|  | Latvia | 6.21% | 4.21% | 9.06% | 1.61% | 1.35% | 1.87% |
|  | Moldova | 4.39% | 2.87% | 6.50% | 1.60% | 1.36% | 1.89% |
|  | Russian Federation | 6.52% | 4.67% | 9.05% | 1.59% | 1.47% | 1.71% |
|  | Ukraine | 3.91% | 2.98% | 5.15% | 1.61% | 1.50% | 1.73% |
| **Europe, Western** | | **4.66%** | **4.28%** | **5.05%** | **1.49%** | **1.40%** | **1.58%** |
|  | Andorra | 6.49% | 4.15% | 9.63% | 1.51% | 1.29% | 1.81% |
|  | Austria | 5.01% | 3.42% | 7.37% | 1.51% | 1.29% | 1.77% |
|  | Belgium | 3.98% | 3.10% | 5.04% | 1.51% | 1.28% | 1.77% |
|  | Switzerland | 6.16% | 4.26% | 8.76% | 1.50% | 1.37% | 1.64% |
|  | Cyprus | 5.75% | 3.89% | 8.21% | 1.51% | 1.28% | 1.77% |
|  | Germany | 4.85% | 4.05% | 5.81% | 1.52% | 1.39% | 1.65% |
|  | Denmark | 5.07% | 3.45% | 7.28% | 1.50% | 1.28% | 1.77% |
|  | Spain | 4.33% | 3.50% | 5.26% | 1.48% | 1.35% | 1.62% |
|  | Finland | 5.98% | 4.91% | 7.15% | 1.65% | 1.51% | 1.79% |
|  | France | 4.80% | 4.03% | 5.65% | 1.47% | 1.35% | 1.60% |
|  | United Kingdom | 3.12% | 2.70% | 3.58% | 1.47% | 1.34% | 1.62% |
|  | Greece | 4.87% | 3.56% | 6.57% | 1.51% | 1.27% | 1.77% |
|  | Ireland | 4.05% | 3.28% | 4.92% | 1.49% | 1.36% | 1.61% |
|  | Iceland | 4.74% | 3.33% | 6.36% | 1.50% | 1.27% | 1.77% |
|  | Israel | 4.58% | 3.49% | 5.73% | 1.50% | 1.30% | 1.74% |
|  | Italy | 4.84% | 3.97% | 5.89% | 1.46% | 1.33% | 1.59% |
|  | Luxembourg | 6.55% | 4.24% | 9.59% | 1.51% | 1.27% | 1.77% |
|  | Malta | 6.58% | 4.28% | 9.68% | 1.50% | 1.26% | 1.78% |
|  | Netherlands | 8.03% | 6.69% | 9.55% | 1.47% | 1.35% | 1.60% |
|  | Norway | 5.94% | 4.63% | 7.57% | 1.47% | 1.35% | 1.60% |
|  | Portugal | 4.32% | 2.98% | 6.11% | 1.51% | 1.28% | 1.77% |
|  | Sweden | 4.76% | 3.31% | 6.75% | 1.50% | 1.26% | 1.75% |

|  | | **MDD** | | | **Dysthymia** | | |
| --- | --- | --- | --- | --- | --- | --- | --- |
| **Region** | **Country** | **Prevalence** | **95% uncertainty interval** | | **Prevalence** | **95% uncertainty interval** | |
| **Latin America, Andean** | | **4.58%** | **3.60%** | **5.81%** | **1.54%** | **1.38%** | **1.71%** |
|  | Bolivia | 3.94% | 2.73% | 5.86% | 1.55% | 1.32% | 1.82% |
|  | Ecuador | 4.38% | 3.04% | 6.26% | 1.54% | 1.31% | 1.81% |
|  | Peru | 4.89% | 3.36% | 7.06% | 1.53% | 1.29% | 1.79% |
| **Latin America, Central** | | **4.40%** | **3.76%** | **5.15%** | **1.50%** | **1.41%** | **1.61%** |
|  | Colombia | 6.31% | 4.27% | 9.02% | 1.51% | 1.29% | 1.77% |
|  | Costa Rica | 4.68% | 3.15% | 6.48% | 1.49% | 1.27% | 1.74% |
|  | Guatemala | 5.35% | 3.65% | 7.56% | 1.51% | 1.28% | 1.79% |
|  | Honduras | 9.22% | 6.83% | 12.23% | 1.50% | 1.28% | 1.77% |
|  | Mexico | 2.96% | 2.30% | 3.78% | 1.50% | 1.42% | 1.58% |
|  | Nicaragua | 5.15% | 3.53% | 7.51% | 1.51% | 1.28% | 1.77% |
|  | Panama | 4.66% | 3.13% | 6.70% | 1.50% | 1.27% | 1.76% |
|  | El Salvador | 5.38% | 3.67% | 7.66% | 1.52% | 1.30% | 1.80% |
|  | Venezuela | 5.06% | 3.48% | 7.07% | 1.51% | 1.27% | 1.77% |
| **Latin America, Southern** | | **4.80%** | **3.65%** | **6.37%** | **1.52%** | **1.35%** | **1.71%** |
|  | Argentina | 5.16% | 3.57% | 7.40% | 1.53% | 1.29% | 1.77% |
|  | Chile | 3.99% | 3.01% | 5.19% | 1.51% | 1.29% | 1.76% |
|  | Uruguay | 4.65% | 3.22% | 6.62% | 1.52% | 1.29% | 1.82% |
| **Latin America, Tropical** | | **5.50%** | **4.39%** | **6.82%** | **1.53%** | **1.44%** | **1.62%** |
|  | Brazil | 5.47% | 4.34% | 6.87% | 1.53% | 1.44% | 1.62% |
|  | Paraguay | 6.39% | 4.16% | 9.63% | 1.53% | 1.30% | 1.77% |
| **North Africa/Middle East** | | **7.35%** | **6.54%** | **8.23%** | **1.53%** | **1.44%** | **1.62%** |
|  | Afghanistan | 22.50% | 17.38% | 29.32% | 1.46% | 1.23% | 1.73% |
|  | United Arab Emirates | 8.12% | 5.45% | 11.54% | 1.36% | 1.15% | 1.62% |
|  | Bahrain | 8.62% | 5.88% | 12.12% | 1.42% | 1.20% | 1.65% |
|  | Algeria | 7.34% | 5.12% | 10.35% | 1.47% | 1.25% | 1.73% |
|  | Egypt | 5.29% | 3.91% | 7.13% | 1.47% | 1.35% | 1.59% |
|  | Iran (Islamic Republic of) | 7.00% | 4.97% | 9.90% | 1.47% | 1.24% | 1.74% |
|  | Iraq | 4.48% | 3.42% | 5.81% | 1.46% | 1.35% | 1.59% |
|  | Jordan | 7.73% | 5.24% | 10.97% | 1.47% | 1.25% | 1.71% |
|  | Kuwait | 7.51% | 5.11% | 10.72% | 1.42% | 1.21% | 1.67% |
|  | Lebanon | 5.27% | 3.90% | 7.06% | 1.49% | 1.37% | 1.61% |
|  | Libya | 9.27% | 6.13% | 13.42% | 1.47% | 1.26% | 1.72% |
|  | Morocco | 6.85% | 4.72% | 9.60% | 1.48% | 1.25% | 1.73% |
|  | Oman | 5.25% | 3.77% | 7.03% | 1.42% | 1.21% | 1.64% |
|  | Occupied Palestinian Territory | 9.01% | 6.01% | 13.31% | 1.47% | 1.24% | 1.74% |
|  | Qatar | 7.99% | 5.31% | 11.78% | 1.35% | 1.15% | 1.58% |
|  | Saudi Arabia | 5.90% | 4.10% | 8.30% | 1.43% | 1.21% | 1.68% |
|  | Syrian Arab Republic | 7.02% | 4.57% | 10.26% | 1.47% | 1.26% | 1.72% |
|  | Tunisia | 7.07% | 4.71% | 10.48% | 1.47% | 1.24% | 1.73% |
|  | Turkey | 6.74% | 5.32% | 8.54% | 1.47% | 1.24% | 1.72% |
|  | Yemen | 7.11% | 4.89% | 9.96% | 1.47% | 1.25% | 1.73% |
| **North America, High Income** | | **4.44%** | **3.76%** | **5.21%** | **1.57%** | **1.46%** | **1.69%** |
|  | Canada | 4.35% | 3.62% | 5.22% | 1.59% | 1.48% | 1.72% |
|  | United States | 4.45% | 3.71% | 5.34% | 1.57% | 1.45% | 1.69% |
| **Oceania** | | **4.72%** | **3.50%** | **6.31%** | **1.62%** | **1.43%** | **1.85%** |
|  | Fiji | 3.46% | 2.26% | 5.07% | 1.61% | 1.36% | 1.89% |
|  | Micronesia (Fed. States of) | 4.36% | 2.96% | 6.21% | 1.62% | 1.38% | 1.91% |
|  | Kiribati | 5.73% | 3.79% | 8.57% | 1.62% | 1.37% | 1.90% |
|  | Marshall Islands | 5.71% | 3.80% | 8.25% | 1.62% | 1.37% | 1.91% |
|  | Papua New Guinea | 5.02% | 3.41% | 7.14% | 1.63% | 1.38% | 1.90% |
|  | Solomon Islands | 3.44% | 2.25% | 5.05% | 1.61% | 1.36% | 1.91% |
|  | Tonga | 4.43% | 3.08% | 6.42% | 1.63% | 1.37% | 1.91% |

|  | | **MDD** | | | **Dysthymia** | | |
| --- | --- | --- | --- | --- | --- | --- | --- |
| **Region** | **Country** | **Prevalence** | **95% uncertainty interval** | | **Prevalence** | **95% uncertainty interval** | |
| **Oceania** |  | **4.72%** | **3.50%** | **6.31%** | **1.62%** | **1.43%** | **1.85%** |
|  | Vanuatu | 4.40% | 2.94% | 6.25% | 1.61% | 1.37% | 1.92% |
|  | Samoa | 4.37% | 2.94% | 6.47% | 1.62% | 1.37% | 1.91% |
| **Sub-Saharan Africa, Central** | | **5.70%** | **4.41%** | **7.31%** | **1.59%** | **1.42%** | **1.78%** |
|  | Angola | 5.03% | 3.41% | 7.09% | 1.59% | 1.35% | 1.87% |
|  | CAF | 5.71% | 3.92% | 8.53% | 1.58% | 1.34% | 1.86% |
|  | Congo, Dem. Rep. | 5.79% | 3.97% | 8.15% | 1.59% | 1.35% | 1.85% |
|  | Congo | 6.45% | 4.45% | 9.21% | 1.58% | 1.35% | 1.84% |
|  | Gabon | 7.20% | 4.93% | 10.31% | 1.58% | 1.34% | 1.87% |
|  | Equatorial Guinea | 7.05% | 4.79% | 10.32% | 1.57% | 1.33% | 1.84% |
| **Sub-Saharan Africa, East** | | **5.43%** | **4.81%** | **6.16%** | **1.56%** | **1.46%** | **1.66%** |
|  | Burundi | 6.06% | 4.25% | 8.53% | 1.57% | 1.33% | 1.87% |
|  | Comoros | 5.78% | 3.94% | 8.40% | 1.56% | 1.32% | 1.84% |
|  | Djibouti | 6.70% | 4.58% | 9.62% | 1.57% | 1.34% | 1.83% |
|  | Eritrea | 6.61% | 4.51% | 9.73% | 1.58% | 1.33% | 1.86% |
|  | Ethiopia | 3.61% | 2.65% | 4.75% | 1.56% | 1.47% | 1.64% |
|  | Kenya | 5.15% | 3.61% | 7.22% | 1.56% | 1.31% | 1.85% |
|  | Madagascar | 5.11% | 3.54% | 7.39% | 1.57% | 1.33% | 1.85% |
|  | Mozambique | 4.58% | 3.03% | 6.51% | 1.58% | 1.34% | 1.86% |
|  | Malawi | 5.77% | 3.85% | 8.41% | 1.56% | 1.32% | 1.87% |
|  | Rwanda | 7.31% | 5.43% | 9.77% | 1.57% | 1.31% | 1.83% |
|  | Sudan | 7.09% | 5.19% | 9.38% | 1.56% | 1.33% | 1.82% |
|  | Somalia | 6.34% | 4.40% | 9.08% | 1.56% | 1.31% | 1.82% |
|  | Tanzania | 6.35% | 4.32% | 9.25% | 1.57% | 1.33% | 1.82% |
|  | Uganda | 6.35% | 4.84% | 8.31% | 1.56% | 1.33% | 1.82% |
|  | Zambia | 5.80% | 3.86% | 8.65% | 1.57% | 1.34% | 1.84% |
| **Sub-Saharan Africa, Southern** | | **5.01%** | **3.96%** | **6.33%** | **1.59%** | **1.49%** | **1.69%** |
|  | Botswana | 7.42% | 4.78% | 10.90% | 1.59% | 1.35% | 1.87% |
|  | Lesotho | 6.28% | 4.38% | 8.79% | 1.61% | 1.36% | 1.88% |
|  | Namibia | 5.00% | 3.38% | 7.16% | 1.60% | 1.34% | 1.87% |
|  | Swaziland | 5.76% | 3.79% | 8.47% | 1.61% | 1.36% | 1.87% |
|  | South Africa | 4.55% | 3.38% | 6.06% | 1.59% | 1.50% | 1.68% |
|  | Zimbabwe | 6.50% | 4.35% | 9.54% | 1.60% | 1.34% | 1.89% |
| **Sub-Saharan Africa, West** | | **4.18%** | **3.69%** | **4.72%** | **1.53%** | **1.44%** | **1.62%** |
|  | Benin | 3.92% | 2.61% | 5.69% | 1.54% | 1.30% | 1.80% |
|  | Burkina Faso | 3.95% | 2.58% | 5.93% | 1.55% | 1.32% | 1.83% |
|  | Cote d'Ivoire | 5.05% | 3.45% | 7.16% | 1.52% | 1.30% | 1.78% |
|  | Cameroon | 4.39% | 2.99% | 6.24% | 1.53% | 1.28% | 1.80% |
|  | Cape Verde | 5.02% | 3.32% | 7.58% | 1.54% | 1.30% | 1.80% |
|  | Ghana | 4.38% | 2.93% | 6.30% | 1.52% | 1.28% | 1.79% |
|  | Guinea | 4.45% | 3.07% | 6.13% | 1.53% | 1.31% | 1.81% |
|  | Gambia | 5.07% | 3.66% | 6.87% | 1.54% | 1.30% | 1.82% |
|  | Guinea-Bissau | 3.91% | 2.53% | 5.62% | 1.53% | 1.30% | 1.81% |
|  | Liberia | 4.60% | 3.11% | 6.65% | 1.54% | 1.30% | 1.80% |
|  | Mali | 5.72% | 3.79% | 8.35% | 1.54% | 1.31% | 1.81% |
|  | Mauritania | 4.99% | 3.48% | 7.01% | 1.54% | 1.31% | 1.80% |
|  | Niger | 4.38% | 3.08% | 6.20% | 1.53% | 1.30% | 1.79% |
|  | Nigeria | 3.69% | 2.95% | 4.65% | 1.52% | 1.44% | 1.61% |
|  | Senegal | 4.47% | 3.01% | 6.38% | 1.54% | 1.30% | 1.81% |
|  | Sierra Leone | 5.65% | 3.90% | 8.01% | 1.54% | 1.31% | 1.80% |
|  | Sao Tome and Principe | 6.51% | 4.35% | 9.46% | 1.54% | 1.32% | 1.82% |
|  | Chad | 5.16% | 3.49% | 7.32% | 1.54% | 1.30% | 1.80% |
|  | Togo | 4.49% | 3.10% | 6.23% | 1.54% | 1.30% | 1.80% |
