# Supplementary material for: Burden of Depressive Disorders by Country, Sex, Age, and Year: Findings from the Global Burden of Disease Study 2010
Source: PLoS Med. 2013 Nov 5;10(11):e1001547. doi: 10.1371/journal.pmed.1001547 (PMC3818162; doi:10.1371/journal.pmed.1001547)
Supplement: Table S3 — Regional DALY and YLD rankings with 95% uncertainty intervals for depressive disorders in 1990. (DOCX) [file pmed.1001547.s005.docx]

**Table S3. Regional DALY and YLD rankings with 95% uncertainty intervals for depressive disorders in 1990.**

|  | **YLDs** | | | | **DALYs** | | | |
| --- | --- | --- | --- | --- | --- | --- | --- | --- |
|  | **MDD** | | **Dysthymia** | | **MDD** | | **Dysthymia** | |
|  | **Order** | **Mean Rank (95% UI)** | **Order** | **Mean Rank**  **(95% UI)** | **Order** | **Mean Rank**  **(95% UI)** | **Order** | **Mean Rank**  **(95% UI)** |
| **Global** | **2** | **2.2 (1-3)** | **19** | **19.5 (12-27)** | **15** | **15.2 (11-18)** | **59** | **58.6 (48-69.5)** |
| Asia Pacific, High Income | 4 | 3.2 (2-5) | 20 | 19.3 (13-27) | 10 | 10.0 (6-15.5) | 37 | 37.8 (29-51) |
| Asia Central | 2 | 1.8 (1-3) | 20 | 20.4 (14-28.5) | 10 | 10.2 (8-13) | 49 | 49.2 (40-62) |
| Asia East | 2 | 2.0 (1-3) | 20 | 17.3 (9-24) | 12 | 12.6 (6-19.5) | 44 | 42.2 (30.5-55) |
| Asia South | 3 | 3.1 (2-4) | 24 | 23 (13-34) | 20 | 20.4 (13-27) | 62 | 61.7 (48.5-79) |
| Asia Southeast | 2 | 1.8 (1-3) | 18 | 19 (10-30) | 11 | 10.8 (6-16.5) | 50 | 51.5 (43-64) |
| Australasia | 2 | 2.8 (2-7) | 21 | 20.3 (14-27) | 7 | 8.2 (5-15) | 37 | 37.6 (25.5-52) |
| Caribbean | 2 | 1.6 (1-3) | 23 | 22.1 (15-29) | 7 | 10 (7-15) | 58 | 55.5 (43-66) |
| Europe Central | 2 | 2.0 (2-2) | 21 | 19.6 (14-26.5) | 7 | 6.7 (4-10) | 46 | 43.6 (30-57) |
| Europe Eastern | 2 | 1.7 (1-2) | 20 | 19.4 (14-26.5) | 5 | 5.3 (3-9) | 46 | 45.6 (36-58) |
| Europe Western | 2 | 2.1 (2-3) | 20 | 20.0 (14-26) | 5 | 5.2 (4-8.5) | 37 | 28.5 (29-54) |
| Latin America, Andean | 1 | 1.6 (1-3) | 24 | 23.2 (16-32) | 10 | 10.3 (5-16) | 50 | 50.6 (42-65) |
| Latin America, Central | 1 | 1.1 (1-2) | 20 | 20.2 (13-30) | 8 | 8.4 (5-12) | 48 | 46.6 (37-59) |
| Latin America, Southern | 2 | 1.6 (1-3) | 19 | 20.6 (14-28) | 7 | 5.9 (3-9) | 49 | 50.5 (38-63) |
| Latin America, Tropical | 2 | 1.8 (1-3) | 22 | 21.0 (13-28) | 10 | 9.7 (7-13) | 47 | 47.6 (40-58) |
| North Africa/Middle East | 2 | 1.9 (1-3) | 23 | 22.3 (16-31) | 8 | 8.4 (7-10) | 53 | 53.7 (43-68) |
| North America, High Income | 2 | 2.7 (1-5) | 20 | 19.4 (13-27) | 8 | 7.5 (3-11) | 39 | 39.3 (31-50) |
| Oceania | 2 | 2.0 (1-4) | 25 | 23.7 (16-34) | 17 | 18.7 (10-28) | 65 | 64.5 (53-77) |
| Sub-Saharan Africa, Central | 2 | 2.0 (1-3) | 30 | 28.5 (18-38) | 22 | 22.1 (16-27) | 70 | 67.2 (56-80) |
| Sub-Saharan Africa, East | 2 | 2.2 (1-3) | 23 | 23.9 (16-37) | 21 | 21.1 (17-26) | 66 | 66.4 (54-81.5) |
| Sub-Saharan Africa Southern | 2 | 2.0 (1-4) | 23 | 23.1 (14-34) | 11 | 12.2 (7-18) | 55 | 55.1 (45-69) |
| Sub-Saharan Africa, West | 3 | 2.8 (2-4) | 29 | 26.9 (18-36) | 24 | 23.0 (17-27) | 69 | 66.2 (55-78) |

*Note. YLDs: years of life lived with disability; DALYs: Disability adjusted life years; MDD: Major depressive disorder; 95% UI: 95% uncertainty interval; Mean Rank: YLD and DALY ranks were estimated for MDD and dysthymia then simulated 1000 times to estimate 95% uncertainty ranges.* The 95% bounds of uncertainty represent the 25^th^ and 975^th^ value of the 1000 draws*; Order: Regional YLDs and DALYs for MDD and dysthymia were ordered* by their mean rank across 1000 draws.
